# Role of Natural Compounds Modulating Heme Catabolic Pathway in Gut, Liver, Cardiovascular, and Brain Diseases

**DOI:** 10.3390/biom14010063

**Published:** 2024-01-02

**Authors:** Sri Jayanti, Libor Vitek, Camilla Dalla Verde, John Paul Llido, Caecilia Sukowati, Claudio Tiribelli, Silvia Gazzin

**Affiliations:** 1 Liver brain Unit “Rita Moretti”, Fondazione Italiana Fegato-Onlus, Bldg. Q, AREA Science Park, ss14, Km 163,5, Basovizza, 34149 Trieste, Italy or srij001@brin.go.id (S.J.); camilla.dallaverde@fegato.it (C.D.V.); johnpaul.llido@fegato.it (J.P.L.); or caecilia.sukowati@brin.go.id (C.S.); ctliver@fegato.it (C.T.); 2 Eijkman Research Centre for Molecular Biology, Research Organization for Health, National Research and Innovation Agency, Cibinong 16915, Indonesia; 3 Institute of Medical Biochemistry and Laboratory Diagnostics, and 4th Department of Internal Medicine, General University Hospital and 1st Faculty of Medicine, Charles University, 12000 Prague, Czech Republic; vitek@cesnet.cz; 4 Department of Life Sciences, University of Trieste, 34139 Trieste, Italy; 5 Department of Science and Technology, Philippine Council for Health Research and Development, Bicutan, Taguig City 1631, Philippines

**Keywords:** bilirubin, herbal medicine, NRF2, heme-oxygenase, MAFLD, neurodegeneration, nutraceuticals, Alzheimer’s disease, cancer, Parkinson’s disease

## Abstract

The crucial physiological process of heme breakdown yields biliverdin (BV) and bilirubin (BR) as byproducts. BV, BR, and the enzymes involved in their production (the “yellow players—YP”) are increasingly documented as endogenous modulators of human health. Mildly elevated serum bilirubin concentration has been correlated with a reduced risk of multiple chronic pro-oxidant and pro-inflammatory diseases, especially in the elderly. BR and BV per se have been demonstrated to protect against neurodegenerative diseases, in which heme oxygenase (HMOX), the main enzyme in the production of pigments, is almost always altered. HMOX upregulation has been interpreted as a tentative defense against the ongoing pathologic mechanisms. With the demonstration that multiple cells possess YP, their propensity to be modulated, and their broad spectrum of activity on multiple signaling pathways, the YP have assumed the role of an adjustable system that can promote health in adults. Based on that, there is an ongoing effort to induce their activity as a therapeutic option, and natural compounds are an attractive alternative to the goal, possibly requiring only minimal changes in the life style. We review the most recent evidence of the potential of natural compounds in targeting the YP in the context of the most common pathologic condition of adult and elderly life.

## 1. Introduction

Far from being only a diagnostic marker for liver diseases, mild elevation of the total serum bilirubin (TSB) concentration has been repeatedly correlated with a lower risk of developing chronic diseases typical of adult and elderly life [1,2,3,4,5,6]. The protection is mediated by bilirubin modulatory activity on the cellular redox state, immunity, cellular metabolism, body glucose, insulin balance, etc. Thus, bilirubin is now considered a biomarker of disease resistance, a predictor of all-cause mortality, and a molecule that can promote health in adults. A complex network links the enzymes involved in bilirubin production and multiple signaling pathways that may be targets of pharmacologic induction [2,4,5]. Based on that, there is an ongoing effort to induce their activity as a therapeutic option. Natural compounds are attractive alternatives to the goal, especially in chronic diseases, requiring a lifelong approach.

## 2. The Heme Catabolic Pathway and Metabolic Checkpoints

**Figure 1:**
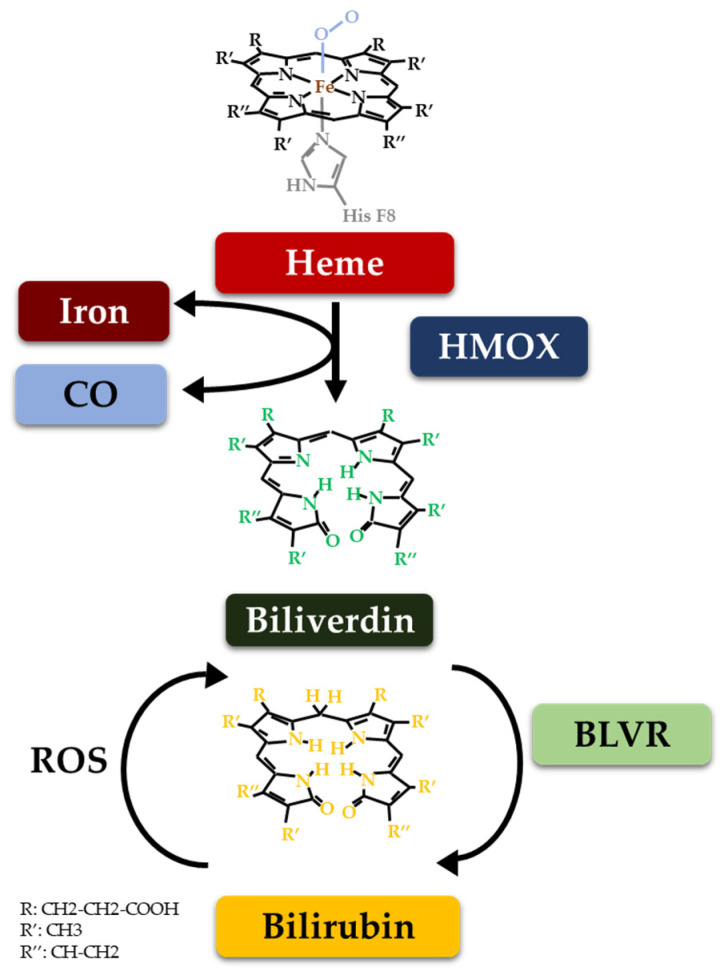
Bilirubin metabolism. CO: carbon monoxide, HMOX: heme oxygenase, ROS: reactive oxygen species, BLVR: biliverdin reductase.

Bilirubin (BR) and its precursor biliverdin (BV) are entirely derived from heme degradation of hemoglobin (Figure 1), which is catabolized by the unique and ubiquitous microsomal enzyme heme oxygenase (HMOX) [7]. Simultaneously, in the reaction catalyzed by HMOX, a molecule of carbon monoxide (CO), a vasoactive and biologically important gaseous molecule, is released together with iron [8]. BV is not present in the circulation due to its rapid conversion to BR by biliverdin reductase (BLVR), a biologically potent enzyme with an important impact on cell function and metabolism [9]. Interestingly, BR has been shown to have even more versatile biological functions in all organs and compartments of the human body, including general antioxidant activities, immunosuppressive functions, and potent cell signaling properties [1,10,11,12,13,14]. HMOX, the key enzyme responsible for the initiation of heme catabolism, has two isoforms (HMOX1, OMIM 141250; and HMOX2, OMIM 141251) [15,16]. HMOX1 is considered the most inducible enzyme in the human body [17,18], and a variety of natural compounds are known as potent inducers of HMOX1 activity [3,19].

The HMOX pathway, through its activation role in gene transcriptions, is closely associated with another evolutionarily conserved cell system, the multifunctional nuclear factor erythroid 2-related factor (NRF2). It is considered not only a regulator of cellular resistance to oxidants, inflammatory stimuli, and toxic xenobiotics, but also a potent modulator of longevity [20,21] and cardiovascular and metabolic diseases [22]. On the other hand, its aberrant activation can increase the risk of development of various diseases, such as diabetes or cancer [23]. Interestingly, also the NRF2 pathway is highly responsive to natural products (see Table 1). Indeed, both stimulatory and inhibitory effects of a wide array of natural products have been reported, with a possible deep impact on the prevention of various civilization diseases [23,24,25,26,27,28,29], including neurodegenerative conditions, such as Alzheimer’s disease (AD), Parkinson’s disease (PD), schizophrenia, multiple sclerosis, amyotrophic lateral sclerosis, perinatal brain injuries, and Duchenne muscular dystrophy, as well as ischemic stroke, CNS trauma, cerebral neoplasms, etc. [5,30,31,32,33,34,35,36,37,38,39,40]. HMOX1 and its products are believed to protect astrocytes and microglia from increased oxidative stress, apoptosis, and inflammation [35,41,42,43] and to promote angiogenesis [44]. The induction of HMOX1 activity by various natural products has become an important therapeutic target for combating certain neurodegenerative and other autoimmune diseases [19,28,45,46,47,48].

It should be mentioned that, after HMOX1 induction, iron is also released [93]. Especially in the brain, excessive iron levels can be toxic due to its pro-oxidant capacity (as example [5,6,44,94,95,96]). On the contrary, HMOX1, iron, cellular redox status, and inflammation regulate the increased vulnerability to ferroptosis in glioblastoma, the most recurrent brain tumor [97,98,99,100,101,102,103].

Also interesting is the observation that BV enhances the expression of CD36 [104], which is involved in fatty acid oxidation and diabetes control [105]. In this regard, it is interesting to note that CD36 has been identified as the aryl hydrocarbon receptor (AhR) target gene [106], with BR being a potent AhR activator [107]. In fact, almost 400 peroxisome proliferator-activated receptors (PPARs) α-dependent genes have been reported to have been significantly modulated by exposure of BV to HepG2 human hepatoblastoma cells [108]. BV also reduces the activation of c-Jun NH2 terminal kinase (JNK), protecting endothelial cells from undergoing apoptosis in vascular injury-induced intimal hyperplasia [109], and has been reported to be protective in cerebral infarction and cerebral ischemia-reperfusion [110,111]. BLVRA activity has significant implications in AD. Its reduction in AD models not only led to increased BACE1 (β-site amyloid precursor protein cleaving enzyme 1) phosphorylation, resulting in higher Aβ deposits, but it also induces insulin resistance by downregulating the insulin receptor (IR) and inhibiting insulin receptor substrate 1 (IRS1) [112]. Meanwhile, the increase of the BLVRA protein level and activity in the brain of the AD model, followed by the increase of UCB, shows a negative (protective) correlation with oxidative stress markers and cognition [113]. BLVRA, which also functions as a transcription factor, interacts with nuclear factor kappa B (NFκB) and leads to the halt of the cell cycle [114]. This results in the downregulation of BLVRA in brain tumors, specifically, meningiomas and gliomas, with implications not yet elucidated with antioxidant status and chemoresistance in these tumor types [6,115]. Moreover, NFkB activation during hypoxic-ischemic injury [116] may contribute to the underlying cause of cerebral palsy [117,118,119,120] and autism spectrum disorders [121,122,123,124,125].

Bilirubin UDP-glucuronosyltransferase (UGT1A1), a phylogenetically old bio-transforming enzyme conjugating bilirubin with glucuronic acid in the liver cell [7], is another important enzyme in the heme catabolic pathway, which is modulated by various natural products [3,126]. *UGT1A1 *expression is substantially under the control of multifunctional nuclear receptors, including the constitutive androstane receptor (CAR), the pregnane X receptor (PXR), the glucocorticoid receptor (GR), the aryl hydrocarbon receptor (AhR), and the hepatocyte nuclear receptor 1α (HNF1α) [127], which regulate *UGT1A1* transcription via the phenobarbital-responsive enhancer module (PBREM) [127].

**Figure 2:**
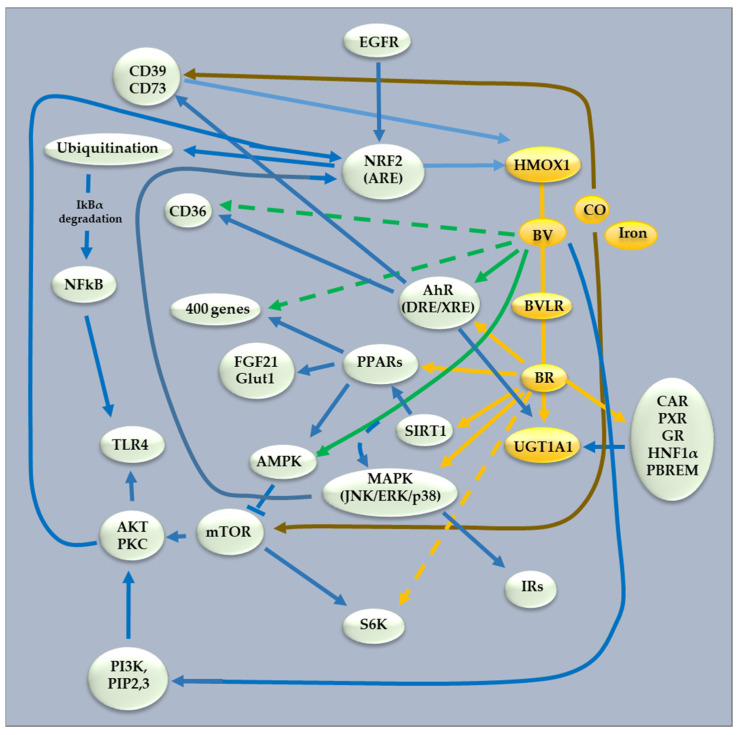
Interconnections between heme metabolism and signaling pathways. Solid line: experimental evidence of a direct link. Dash lines: no experimental evidence of a direct action. Green lines: biliverdin action; yellow lines: bilirubin actinin; brown lines: CO action; blue lines: all other actions/connections. Yellow EFGR: epidermal growth factor receptor; NRF2: nuclear factor erythroid 2–related factor 2; ARE: antioxidant response element; CD: cluster of differentiation; HMOX: heme oxygenase; BV: biliverdin; BVLR: biliverdin reductase; BR: bilirubin; UGT1A1: UDP-glucuronosyltransferase 1A1; CAR: constitutive active/androstane receptor; PXR: pregnane X receptor; GR: glucocorticoid receptor; AhR: aryl hydrocarbon receptor; DRE/XRE: drought/xenobiotic-responsive elements; HNF1: hepatocyte nuclear factor 1α; PBREM: phenobarbital (PB)-responsive enhancer module; PPAR: peroxisome proliferator-activated receptor; SIRT: silent information regulator; MAPK: mitogen-activated protein kinase; JNK: Jun kinase; ERK: extracellular signal-regulated kinase; mTOR: mechanistic Target of Rapamycin; AMPK: AMP-activated protein kinase; AKT: protein kinase B; PKC: protein kinase C; TLR4: toll-Like receptor 4; NFkB: nuclear factor-κB; PIP: phosphatidylinositol biphosphate; PI3K: phosphatidylinositol-3-kinase; IRs: insulin receptor; S6K: protein S6 kinase; FGF: fibroblast growth factor; Glut1: glucose transporter type 1.

Interestingly, BR serves as a ligand for these nuclear receptors, which also contribute to glucose and lipid metabolism [12] and the pathogenesis of cardiovascular diseases and other diseases of civilization [128,129]. Meanwhile, a slightly elevated TSB is negatively correlated with multiple neurologic conditions (for a review on the correlation between TSB and neurologic conditions, see [6]). This strongly supports the idea that the nutraceuticals able to increase bilirubin production might help to prevent neurodegenerative damage [44]. Of relevance, BR has been recently demonstrated to prevent dopaminergic neuron (DOPAn) demise in an ex vivo model of Parkinson’s disease by normalizing TNFα, the determinant in DOPAn death [130]. Remarkably, CAR/PXR/GR/AhR/HNF1α are substantially modulated by dietary components, including natural products [131,132]. It is also important to note that BR per se also serves as a ligand for other metabolic nuclear receptors, such as peroxisome proliferator-activated receptors (PPARs) α and γ, being considered master regulators of cellular and whole-body energy homeostasis, with important roles in the pathogenesis of obesity, diabetes mellitus, metabolic syndrome, and aging [12,133,134,135]. The direct activating effect of bilirubin on PPARα has recently been convincingly demonstrated in various experimental studies (reviewed in [74]). In fact, physiologically relevant BR concentrations were capable of activating PPARα with the same magnitude as that of fenofibrate, a clinically used agonist of this nuclear receptor. In addition, a recent in silico analysis revealed that bilirubin resembles structurally the molecule of fenofibrate [39]. Furthermore, acute treatment of mice with bilirubin resulted in increased expression of hepatic PPARα target genes, including fibroblast growth factor 21 (FGF21) [104]. BR also affects FGF21 [80,81], considered a late-acting fed and fasting-state hormone [136], but interestingly, also an insulin signaling pathway [137,138,139,140] (insulin being considered an immediately acting hormone). BR also affects the expression of PPARγ [91,137], another master regulator of adipogenesis and obesity [141]. Although not all available data are consistent, it seems that modulation of the PPARγ pathway is biologically and clinically relevant. It has been reported that PPARγ agonists activated the AMP-activated protein kinase (AMPK) [77,142,143,144], an important cellular energy sensor [145,146]. Increased AMPK phosphorylation has been reported in subjects with benign hyperbilirubinemia (Gilbert’s syndrome) [77]. PPARγ activation of AMPK also led to inhibition of the mammalian target of rapamycin (mTOR signaling), an evolutionarily conserved nutrient-sensing protein kinase that regulates metabolism, aging processes, and overall life span [147,148], as well as dephosphorylation of a downstream factor, S6K [143]. Similar results on inhibition of S6K phosphorylation were also obtained in our study in human fibroblasts exposed to physiological concentrations of BR [14,149]. Interestingly, dysregulation of AMPK and mTOR hyperactivation was reported in BLVRA-deficient mice [84]; and in another study, BV inhibited TLR4 signaling in leukocytes and triggered phosphorylation of mTOR-specific targets, including Akt, PKC, and AMPK [89], suggesting the importance of BV and BR for modulation of these signaling pathways (Figure 2).

The same effects on the activation of AMPK and mTOR inhibition due to BR were also observed in mice with NAFLD treated with CO, another product of the heme catabolic pathway [85]. PPARγ coactivator- α (PGC1α) is a transcription coactivator of PPARγ that plays a central role in the regulation of cellular energy metabolism [150]. It has been shown that hyperbilirubinemic subjects with Gilbert’s syndrome have substantially lower BMI and serum concentrations of glucose, insulin, C-peptide, and triacylglycerol, the activation of AMPK, PPARα/γ, and peroxisome proliferator-activated receptor-gamma coactivator 1α (PgC1α) being considered the most important factors responsible for these observations [77]. Sirtuins (SIRTs) are a family of signaling proteins involved in metabolic regulation [151]; SIRT1, a deacetylase modulating PPARγ and PGC1α, hence controlling fat and glucose metabolism, is metabolically one of the most important. SIRT1, PGC1α, together with AMPK form an energy-sensing network that controls energy expenditure [152]. Interestingly, BR was demonstrated to upregulate SIRT1 in an experimental NAFLD in vitro study [87]. Furthermore, SIRT1 influences the acetylation of microtubule-associated tau (MAPT) that contributes to the preservation of neuronal cytoskeletal stability important for neuroprotection in cerebral ischemia/reperfusion [153]. All BR-modulated pathways described above are interlinked, as demonstrated by the role of the CD39 ectonucleotidase/adenosine pathway in immunity and inflammation, which is under the control of HMOX1 [154,155], and also have important effects on glucose metabolism and insulin signaling [156], the AMPK pathway [157], and the pathogenesis of liver diseases, such as metabolic dysfunction-associated fatty liver disease (MAFLD) [158,159], indicating the complexity of BR-related modulation of cell signaling. It should be noted that some of the anti-inflammatory effects of CO are mediated via its CD39 ectonucleotidase/adenosine pathway [79]. All of these experimental data fit in with the clinical observation of low PPARα expression, as well as systemic BR concentrations in obese subjects [160]. In addition, BR has also been shown to ameliorate experimental colitis by upregulation of CD39 [78]. The effect of the heme catabolic pathway on cell metabolism is probably even more complex. As proven in a liver-specific BLVRA knockout mouse model [91], BLVRA (and hence bilirubin, its enzymatic product) regulates hepatic lipid metabolism by directly affecting the key enzymes implicated in lipid metabolism. In addition to AMPK, these also include acetyl-CoA carboxylase, an essential and rate-limiting enzyme in fatty acid metabolism [161] and glycogen synthase kinase 3b (GSK3b), one of the most active cellular kinases, with more than 100 known targets, involved in the regulation of multiple cellular functions, including lipid and glucose metabolism, among others [162,163]. Thus, BR appears to act as a multifunctional modulator at multiple cellular metabolic checkpoints, which are often mutually interrelated. This is exemplified by bilirubin-induced suppression of p38 MAPK (but also other MAPKs) [164] in ischemia/reperfusion after heart transplantation, known to be crucial for insulin signaling, as well as atherogenesis [165].

Several natural products have been reported to activate both PPARα and PPARγ nuclear receptors [75,76], but also AMPK [90]. Similarly, a variety of natural products, including curcumin, resveratrol, quercetin, or apigenin, to mention at least some of them, have been shown to inhibit mTOR signaling [86] or activate SIRT1 [88] (summarized in Table 1).

## 3. Natural Compounds with Demonstrated Effects on Bilirubin and Its Metabolic Enzymes

### 3.1. Natural Compounds

#### 3.1.1. Flavonoids

There is strong clinical evidence indicating that treatment with silymarin (a seed extract of milk thistle, *Silybium marianum *(L.) Gartn.) [166] flavonolignans results in mild elevation of TSB concentrations, as reported in silybin-treated patients with prostate cancer [167], as well as hepatitis C [168]. It is important to note that some of the minor silymarin flavonolignans are even more biologically active, as evidenced in our recent experimental study, in which dehydrosilybin A and B were potent partial inhibitors of UGT1A1 activity [166]. Flavonoids are also promising chemoadjuvants for the treatment of neurodegenerative diseases [169]. Fisetin is one of the examples of flavonoid tested with positive results in the experimental model of amyotrophic lateral sclerosis by inducing HMOX1 [170]. A natural aglycone flavonoid from the Erigeron plant, known as breviscapine, decreases neuronal apoptosis by promoting the expression of NRF2, followed by the increase in antioxidant enzymes, including HMOX1, in traumatic brain injury [54]. Eriodictyol, a flavonoid compound that is commonly present in the rinds of citrus fruits and certain traditional Chinese herbal remedies, also exhibits anti-AD’s effect. It ameliorates memory impairment, inhibits Aβ aggregation, and decreases Tau phosphorylation in the brains of AD mice by modulating the Nrf2/HMOX1 signaling pathway [171].

Another compound in this category, luteolin, has also been proposed as a phototherapeutic agent for PD due to its antioxidant, anti-inflammatory, and anti-apoptosis effects. It can enhance the HMOX1 expression and the link between Nrf2 and its antioxidant element, providing a good adaptive survival response against oxidative damage. Furthermore, this compound also inhibits the expression of various pro-inflammatory compounds, after the LPS stimulus, such as inducible NO synthase (iNOS), TNFα, cyclooxygenase 2 (COX2), interleukin 1β (1Lβ), nitric oxide (NO), and prostaglandin E2 [60].

#### 3.1.2. Curcumin

Curcumin is a major polyphenolic compound from the curcuminoid group of phytochemicals, originating from the plant *Curcuma longa*. Curcumin is a potent inducer of HMOX1 with many clinical consequences. Its use as a nutraceutical has been investigated in several studies focused on the prevention of neurotoxicity and neurodegeneration (reviewed in [172]). Despite the small number of participants, a clinical trial of a curcumin formulation with enhanced oral bioavailability showed an improvement in survival probability over a 12-month period in amyotrophic lateral sclerosis [173,174]. In an in vitro model of the disease (microglia cells that have been activated by LPS), curcumin reduces the production of iNOS and NO release by increasing the expression of NRF2 and HMOX1 and downregulating the NFkB signaling pathway, which inhibits the release of pro-inflammatory cytokines IL6, IL1, and TNFα [175]. NFkβ/MAPK pathways have also been involved in curcumin-mediated anti-inflammatory action in microglia [61]. Similarly, in primary astrocytes, curcumin activates NRF2 target genes, including HMOX1 and NQO1 (NAD(P)H dehydrogenase quinone 1); lowers the amount of intracellular ROS; and lessens oxidative damage and mitochondrial dysfunction [176], as well as curcumin; it also induces an improvement of mercury-induced cytotoxicity via the NRF2/ARE/PKCδ pathway [64]. The antioxidant properties of curcumin have also been shown in neurons, by a NRF2/PKCδ-mediated induction of p62 (ubiquitin sensor and selective autophagy receptor) phosphorylation [70]. Although the in vitro neuroprotective action of curcumin (and astragaloside) in depression has been suggested to be mediated by the tyrosine protein kinase (TRK) β/MAPK/PI3K/CREB signaling pathways-induced upregulation of BDNF (brain-derived neurotrophic factor) [177], in in vivo models, the antidepressant properties of curcumin have been found to be involved via AKT1, NRF2, and ARE signaling [65,71]. Furthermore, in vivo, dietary supplementation with curcumin has been shown to reverse the microglial and astrocytes activation via NRF2/TLR4/NFkB/RAGE (receptor for advance glycation end products) signaling in a rodent model ethanol-induced neurotoxicity [68], and acts as an antioxidant in intracerebral hemorrhage, traumatic brain injury, quinoline acid-induced glutamate neurotoxicity, and ischemic injury models via the NRF2/HMOX1/AKT pathways [49,62,63,66,67,72]. Moreover, curcumin also exhibits anti-cancer effects by enhancing HMOX1 expression and activating ferroptosis, a form of oxidative cell death, in thyroid cancer [178].

#### 3.1.3. Astragaloside

Astragaloside IV (AST) is one of the main active ingredients of *Astragalus* membranaceus var. mongholicus, or A. membranaceus, a traditional Chinese herb. AST was found to exhibit neuroprotection in rats with ischemic stroke by upregulating SIRT1 expression, which promotes anti-inflammation and antioxidant production [153]. Similarly, AST reduces LPS-mediated neuroinflammation (microglia) by acting on the activation of NRF2, and HMOX1 via the ERK pathway [57]. AST also improves endothelial dysfunction in cardiovascular disease by activating the NRF2 signaling pathway and promoting the transcription of antioxidant enzymes including HMOX1 [179]. The combination of AST and Panax notoginseng results in protection in cerebral ischemia-reperfusion mice by increasing the nuclear translocation of Nrf2, followed by the increase of HMOX1, with a decrease of oxidative stress markers and an increase of the SOD and GSH level [180].

#### 3.1.4. Vitamins

Vitamin C consumption in combination with vitamin E for one month among AD patients could reduce oxidative stress markers in plasma and CSF (cerebrospinal fluid) [181]. Vitamin C indeed promotes antioxidant activity by activating NRF2 and increasing HMOX1 expressions [182]. It also acts against inflammation by decreasing TNFα and IL6 and increasing IL10 [183]. There is growing evidence that vitamin D deficiency can affect brain processes, including cognition, and significantly increase the risk of AD [184], speeding up the aging process through the disruption of redox cell signaling pathways [184]. In line, maxacalcitol, a vitamin D analogue, substantially lowered neuroinflammation and enhanced expression of NRF2 and its downstream effectors (HMOX1 and GSH) in the animal model for AD [185].

#### 3.1.5. Madecassoside

The Indian Ayurvedic system of medicine has utilized *Centella asiatica* to improve neurocognitive functioning. The most abundant triterpenoid saponin isolated from the plant, madecassoside, protects the neurons from apoptosis due to free radicals by increasing the antioxidant activity in the ALS mouse model. It has also been reported to improve LPS-mediated neurotoxicity in rats with its anti-inflammatory activities and activation of the Keap1-Nrf2/HMOX1 signaling pathway [186].

#### 3.1.6. Green Tea

The clinical study by Arab et al. showed that drinking green tea (leaf of *Camellia sinensis*) 2 g/day for two months has a positive impact on cognitive performance in severe AD patients by increasing the total antioxidant capacity by around 20% [187]. Additionally, epigallocatechin-3-gallate (EGCG), a bioactive compound of green tea, has been demonstrated to stimulate antioxidant defense mechanisms by activating the Nrf2/ARE pathway and antioxidant enzymes by activating AKT and ERK1/2 pathways [188]. In particular, EGCG functions as an antioxidant by modulating neurodegenerative inflammatory processes, such as ferroptosis and microglia-induced cytotoxicity, and by activating signaling pathways important for neuronal survival [174].

#### 3.1.7. S-Allyl Cysteine

S-allyl cysteine, a water-soluble organosulfur compound containing an amino acid isolated from the garlic bulb (*Allium sativum*), ameliorates cognitive deficits in streptozotocin-diabetic rats via suppression of oxidative stress, inflammation acting on the NRF2- HMOX1, TLR4, and NFκB signaling cascade [58].

#### 3.1.8. 20C

20C, a bibenzyl compound isolated from *Gastrodia elata *(a commonly used traditional Chinese medicine for therapeutic applications, like epilepsy and vertigo treatment), protects PC12 neurons from rotenone-induced lesions mimicking PD in vitro by normalizing (increasing) HMOX1 and NQO1 mRNA and the protein expression level, and by reverting ROS accumulation, cytochrome c release, and apoptosis via NRF2 signaling [47].

#### 3.1.9. *Achyranthes bidentata*

The extract *Achyrantes bidentata* polypeptide K (ABPK) from the *Amaranthaceae *family, another Chinese herbal medicine, reduces inflammation in LPS-challenged microglia (BV2 cells, NO, IL6, TNFα, PGF2) via NRF2/HMOX1 signaling [43].

#### 3.1.10. *Coriolus versicolor* and *Hericium erinaceus*

Consumption of certain mushrooms affects neurocognitive functioning. Two of the widely studied medicinal mushrooms are *Coriolus versicolor *and *Hericium erinaceus. *The combined extracts of these two mushrooms lower LXA4, a metabolic product of arachidonic acid, which is considered an endogenous “stop signal” for inflammation, astrocytes and microglia activation, α-synuclein content, apoptosis, and dopaminergic neuron death, finally improving the motor abilities in a rodent model of PD [48].

#### 3.1.11. Hyperoside (Quercetin 3-O-galactoside)

Hyperoside (quercetin 3-O-galactoside) from *Acer tegmentosum*, a Korean traditional medicine, has demonstrated antioxidant action in cultured neurons serving as an in vitro model of PD via NRF2/HMOX1 [52].

#### 3.1.12. Acerogin A

Acerogenin A, a natural compound from *Acer nikoense* Maxim used in Japanese folk medicine, has been shown to prevent glutamate neurotoxicity in vitro by acting through NRF2/HMOX1 and PIK3/Akt signaling, with glutamate neurotoxicity being a common pathological mechanism in many neurodegenerative and neurologic conditions [55].

#### 3.1.13. Kaempferol, Ginsenoside rh2

Kaempferol and ginsenoside rh2, the most active principle of the Kaixinsan formula, used as an antidepressant in traditional Chinese medicine, are effective in reverting the pro-oxidant cellular status of SH-SY5Y cells exposed to H_2_O_2_ by increasing thioredoxin reductase activity via NRF2/AKT [71].

#### 3.1.14. Mangiferin

Mangiferin is a natural compound originating from multiple plants, including *Mangifera indica *L. Through recent studies, it has been demonstrated to have an important role in protecting neurons from degeneration. In fact, it has some crucial antioxidant properties by preventing the formation of hydroxyl radicals and ROS [59]. Mangiferin has protective effects on PD in vitro and in vivo models by enhancing antioxidant defense, including the expression of NRF2 and HMOX1 [189]. Mangiferin also can downregulate NFκB in breast cancer, followed by increased apoptosis as one of the consequential effects [190].

#### 3.1.15. Ellagic Acid

Ellagic acid is a chromene-dione derivative (C_14_H_6_O_8_) present in many fruits and nuts, such as pomegranates, black raspberries, raspberries, strawberries, walnuts, and almonds. It is a phenolic antioxidant with higher antioxidant activity than vitamin E, succinate, and melatonin. It can also inhibit the major pro-inflammatory pathways, such as NFkB, MPAKs, and JAK/STAT, preventing inflammatory molecules’ release (e.g., TNFα, IL1β, IL6, and iNOX) [69].

### 3.2. Natural Compounds Mimicking Products of the Heme Catabolic Pathway

#### 3.2.1. Tetrapyrroles from *Spirulina platensis*, Phycocyanin, and Phycocyanobilin

*Spirulina (Arthrospira) platensis* is a blue-green freshwater alga widely used as a dietary supplement. It is rich in proteins, carotenoids, essential fatty acids, vitamin B complex, vitamin E, and minerals, such as copper, manganese, magnesium, iron, selenium, and zinc. It is a source of potent antioxidants, including spirulans (sulfated polysaccharides), seleno compounds, phenolic compounds, and phycobiliproteins (C-phycocyanin and allophycocyanin) [191]. Numerous studies have demonstrated that dietary supplementation of *S. platensis* is helpful in the prevention and treatment of atherosclerosis, diabetes, and/or cancers (for review see [192]). C-phycocyanin is a light-harvesting biliprotein that is possibly implicated in the biological effects of *S. platensis*. C-phycocyanin contains a covalently linked chromophore called phycocyanobilin (PCB) [193], a linear tetrapyrrolic molecule structurally similar to BV that constitutes up to 1% of the dry weight of *Spirulina*. Interestingly, phycocyanobilin (PCB) can be metabolized by BLVR to phycocyanorubin, similarly as BV is reduced to BR in the human body [194]. Therefore, *S. platensis* appears to be a rich nutraceutical source of PCB [195], which is known to significantly inhibit nicotinamide adenine dinucleotide phosphate (NADPH) oxidase activity, activate the action of antioxidant enzymes, and have anti-inflammatory and cell signaling activities with an expected substantial impact on human health, including fighting the neurologic adverse effects of COVID-19 [73,196,197].

We have shown that *S. platensis* and PCB markedly increased Hmox1 in experimental models of atherosclerosis [198]. Therefore, activation of HMOX1 and the heme catabolic pathway appears to be an important mechanism of this food supplement for the reduction of atherosclerotic diseases. Due to their antioxidant effects, C-phycocyanin and PCB (but also BR and BV) protected diabetic db/db mice against albuminuria and renal mesangial expansion in db/db mice, and normalized tumor growth factor-β and fibronectin expression, suggesting a novel and feasible therapeutic approach to prevent diabetic nephropathy [199]. Importantly, antidiabetic effects of *S. platensis*, C-phycocyanin, and PCB were reported in diabetic male albino rats treated with streptozotocin, with decreases of blood glucose concentrations, improvement of insulin resistance and blood lipids levels, and restoration of pancreatic cell morphology [200].

The anticancer effects of *S. platensis* and its tetrapyrrolic components (PCB and chlorophyllin, a surrogate molecule for chlorophyll A) in biologically relevant doses were proved on experimental pancreatic cancer models in another study. These models included nude mice xenotransplanted with pancreatic cancer cells, suggesting a chemopreventive role of this edible alga, whose dietary supplementation with this alga might enhance the systemic pool of linear tetrapyrroles mimicking BR [201]. Similarly, in an experimental in vitro study by Hussein et al., C-phycocyanin and PCB treatment led to potent antiproliferative, and pro-apoptotic effects in MCF-7 breast cancer cells [202].

PCB also showed strong anti-inflammatory and general hepatoprotective effects in mice with CCl4-induced liver injury, with a marked improvement inf the survival rate of acute liver failure in mice injected with a lethal dose of CCl4 [203].

Importantly, *Spirulina alga* and its associated bioactive compounds appear to also be important in chemoprevention of neurodegenerative diseases [73,204]. Activated microglia, displaying NADPH oxidase activity, are believed to contribute substantially to the pathogenesis of many brain diseases, such as PD and AD, and multiple sclerosis, but also cerebral ischemia [205,206,207] or COVID-19-induced damage of the nervous system [73]. Although direct clinical evidence is lacking, experimental data suggest that *S. platensis* extracts could ameliorate the risks of these diseases [208]. A diet high in *Spirulina* ameliorates the loss of dopaminergic neurons in the mouse model of PD [209]. Furthermore, PCB improved the neurological outcomes in a mouse model of experimental autoimmune encephalitis (EAE) that mimics multiple sclerosis conditions, with significant inhibitory effects on pro-inflammatory cytokines. Interestingly, a reduction of demyelination, active microglia/macrophages density, and axonal damage was detected, along with an increase in oligodendrocyte precursor cells and mature oligodendrocytes, when assessing the spinal cords of EAE mice treated with PCB. Due to the increased treatment effects of PCB when used with IFNβ therapy, PCB was suggested for use with IFNβ as a disease-modifying agent for multiple sclerosis [210]. Similar results were found also in other EAE experimental studies, demonstrating a clear tendency for amelioration of the clinical severity of the disease promoted by the treatment with PCB in an EAE model with a reduction in the levels of the pro-inflammatory cytokines IL17, IFNγ, and IL-6 in the brains of animals treated with PCB. Similar observations were also obtained in animal models of cerebral ischemia [205,211]. The demyelination potential of C-phycocyanin and PCB with their possible clinical use in patients with demyelinization disorders has been reviewed recently [207].

PCB also was shown to have potential inhibitor activity against main protease (Mpro) and papain-like protease (PLpro) of human and animal coronaviruses, indicating broad-spectrum inhibitor activity of PCB. In addition, in silico studies with the Mpro and PLpro enzymes revealed that other tetrapyrrolic phycobilins, such as phycourobilin and phycoerythrobilin, will have similar binding affinity to SARS-CoV-2 Mpro and PLpro [212]. Hence, PCB and *S. platensis* were suggested as promising nutraceuticals against COVID-19-induced brain damage [73]. In addition, antiviral activities of *S. platensis-*derived compounds were shown in a study by Chen et al., who reported inhibitory effects of Spirulina extract against influenza virus replication and reduction of virus-induced mortality [213]. In in vitro studies, antiviral activities of *S. platensis *extract were also reported against herpetic viruses, measles, and mumps viruses, as well as human immunodeficiency virus (for a review, see [214]). Interestingly, in a small clinical study, *S. platensis* consumption for 12 months improved the immunological profile of HIV-infected patients [215]. It should be noted that in countries with high algae consumption, such as Japan, Korea, or Chad, the prevalence rate of HIV infection and AIDS remains substantially lower compared to other countries [214,216].

Due to the potent antioxidant, antibacterial, and other beneficial biological effects, extracts from *S. platensis* exhibits have been used in skincare formulations for the treatment of acne [217], sunscreens (*S. platensis* having a very high sun protecting factor, SPF) [218], face masks lip balms, and ointments for their wound-healing and anti-aging properties [219]. Therefore, the global market is estimated to be as much as USD 2000 million by 2026, with an overall *S. platensis* consumption of more than 321,000 tons [220].

#### 3.2.2. Artificial Bezoar

BR is present also in pulverized bovine gallstones (*Calculus bovis*, artificial bezoar, *Niu Huang* in Chinese) used for centuries for a variety of human diseases in traditional Chinese medicine [221,222]. Preparations of these pulverized gallstones have a high content of BR of no less than 25% by weight [222].

Neuroprotective effects of artificial bezoar were reported in an experimental study on male Sprague-Dawley rats with induced cerebral ischemia. Pretreatment with An-Gong-Niu-Huang Wan, a complex traditional Chinese medicine formulation containing Calculus bovis, significantly ameliorated ischemic damage to the brain in a dose-dependent manner, including a reduction in the neurological deficit score and infarct area [223]. Other reported activities of *Calculus bovis* on the nervous system include sedative, anticonvulsant, analgesic, antiepileptic, or even anti-schizophrenic (reviewed in [222]). Furthermore, *Calculus bovi*s was found to also have protective effects on the heart, vessels, lungs, or liver [222], or even experimental cancer, as shown for experimental breast cancer, human hepatoblastoma [224].

#### 3.2.3. Chlorophylls

Chlorophyll is a tetrapyrrolic compound structurally related to BR [7] that is likely to exert similar protective biological activities. Dietary intake of green leafy vegetables rich in chlorophyll is associated with protection against cancer and other civilization diseases, including neurodegenerative diseases [50,225]. Although their resorption from the gut lumen is not high, chlorophylls appear to be important for their potential systemic cancer-preventive effects [226], reaching biologically relevant concentrations even in peripheral tissues [227]. Anti-proliferative and antioxidant effects of chlorophylls (chlorophyll a/b, chlorophyllin, and pheophytin a) were reported recently in our experimental in vitro and in vivo pancreatic cancer study [228].

## 4. Natural Compounds Targeting the Heme Catabolic Pathway

As described in detail in our recent review [14], targeting the individual parts of the heme catabolic pathway represents an important way to increase tissue or systemic levels of BR.

### 4.1. Modulation of HMOX1

As emphasized above, HMOX1 is inducible by a variety of natural products and is an important chemotherapeutic target for the prevention and treatment of many neurodegenerative and other autoimmune diseases [19,45,46]. These natural HMOX1 modulators include various polyphenolic compounds found in plants, such as curcuminoids; flavonoids, such as quercetin, EGCC, genistein, eriodictyol, or certain flavonolignans of silymarin complex, caffeic acid, and resveratrol; and natural coumarins, such as esculetin or fraxetin [3,229,230].

### 4.2. Modulation of BLVRA

BLVRA induction seems always beneficial, while its enzymatic inactivation looks detrimental, possibly by reducing the final concentration of unconjugated bilirubin (UCB) inside the cell [9]. Interestingly, BLVRA expression is also inducible by natural products, leading to increased production of BR, as demonstrated for Korean red ginseng in an in vitro study in murine hippocampal astrocytes improving mitochondrial functions via the LKB1-SIRT1-ERRα axis [231].

### 4.3. Modulation of the Hepatic Transport of Bilirubin

Inhibition of organic anion transporter 1B1 (OATP1B1), a transporter crucial for UCB uptake in the liver, by compounds or substances relying on this transporter for transport could lead to a substantial increase in bilirubin concentrations [3]. Basolateral uptake of bilirubin in liver cells is another druggable target to increase mild serum BR concentrations. In fact, interference with OATP1B1 bilirubin transporter was reported for many natural compounds commonly present in certain foods, food supplements, and herbs [3,232].

### 4.4. UGT1A1 Modulation

As UGT1A1 is responsible for intrahepatic BR conjugation, its suppression will lead to an increase in BR level [3]. Many natural compounds, often used as nutraceuticals, have UGT1A1-suppressing activity resulting in mild elevation of systemic BR concentrations, including silymarin flavonolignans or EGCC, or many Japanese and Chinese herbs commonly used in traditional herbal medicine [3].

### 4.5. Gut Microbiome Modulation

By affecting the enterohepatic and enterosystemic circulation of BR [233], BR-reducing bacteria in the gut lumen can affect systemic concentrations of the pigment [234]. Although no experimental and clinical data on possible modulation of gut microbiome metabolizing bilirubin are available today, likely, certain probiotics or other modulating agents capable of affecting intestinal metabolism of bilirubin with possible beneficial clinical consequences will become available [235].

## 5. Conclusions

The beneficial potential of bilirubin in human health is clear in large measure. The modulability of HMOX1 and its metabolic checkpoints by natural compounds makes this system a great and feasible target to combat various public health concerns and age-related diseases with minimal changes in the diet. Further studies, and collaborations with the food or pharma-nutraceutical industry, are needed to specify all this solid information in a varied and easy-to-take functionalized alimentary regimen to promote health day by day.

## Figures and Tables

**Table 1:** Metabolic checkpoints affected by natural compounds and also by the heme catabolic pathway. NRF2, nuclear factor erythroid 2—related factor 2; HMOX, heme oxygenase; PI3K, phosphoinositide 3-kinase; AKT, serine/threonine kinase; EGFR, epidermal growth factor receptor; ERK, extracellular signal-regulated kinase; ROS, reactive oxygen species; NQO1, NAD(P)H: Quinone Acceptor Oxidoreductase Type 1; NO, nitric oxide; TNFα, tumor necrosis factor alpha; IL, interleukin; SOD, superoxide dismutase; GSH, glutathione; PERK, protein kinase R (PKR)-like endoplasmic reticulum kinase; NFkB, nuclear factor k-light-chain-enhancer of activated B cells; TLR4, toll like receptor 4; MAPK, mitogen-activated protein kinases; PKC, protein kinase C; GST, glutathione g-transferases; p62, selective autophagy receptor p62; AKT, protein kinase B (PKB), also known as Akt; NADPH, nicotinamide adenine dinucleotide phosphate; PPAR, peroxisome proliferator-activated receptor; PGC1α, peroxisome proliferator-activated receptor-α coactivator-1α; CD, cluster of differentiation; CO, carbon monoxide; FGF21, fibroblast growth hormone 21; mTOR, mammalian target of rapamycin; SIRT1, NAD-dependent deacetylase sirtuin-1; AMPK, AMP-activated protein kinase; T2DM, type 2 diabetes; GSK3b, glycogen synthase kinase-3b; BLVRA, biliverdin reductase A.

Metabolic Checkpoint	Heme Catabolic Pathway Modulator	Natural Compound (Some Examples)	Possible Clinical Impact
NRF2	NRF2 activates HMOX1 [29,49,50] Bilirubin activates NRF2 [51]	Sulphoraphan, curcumin, bixin, apigenin, cinnamaldehyde, withaferin A, luteolin, wogonin, chrysin… [23,24,25,26,27,28]	Regulator of cellular resistance to oxidants, inflammatory stimuli and toxic xenobiotics, modulator of longevity and cardiovascular and metabolic diseases.
	HMOX1	20C (bibenzyl compound isolated from Gastrodia elvata) [47]	Suppresses the pro-apoptotic effect of Rot by inhibition of Bax and suppress the accumulation of intracellular ROS and the collapse of the mitochondrial membrane potential.
	HMOX1	(ABPK) achyrantes bidentata polypeptide K [43]	Neuroprotective agent inhibiting the neuroinflammation on BV2 microglia cell culture.
	HMOX1	Coriolus versicolor, Hericium erinaceus [48]	Anti-inflammatory modulating the lipoxin A4 levels (LXA4), resolving neuroinflammation and limiting the motor and non-motor symptoms, typical of PD.
	HMOX1	Hyperoside (quercetin 3-O-galactoside) [52]	Protects cultured dopaminergic neurons from death via ROS-dependent mechanisms.
	HMOX1	Berberine (BBR) [53]	Binds specific DNA sequences triggering DNA repair process.
	HMOX1	Breviscapine [54]	HMOX1 and NQO1 increases.
	HMOX1 via PI3K/AKT	Acerogin A [55]	Prevent glutamate-induced oxidative damage.
	HMOX1 via EGFR/ERK	Astragaloside IV+/− Panax notoginseng [56,57]	Reduction of the oxidative stress markers, inhibition inflammatory mediators (NO, TNFα, IL6) and increase of SOD and GSH level.
	HMOX1 and NFkB/TLR4 signaling cascade	S-allyl cysteine (SAC) from aged garlic extract [58]	Improve cognitive deficits by attenuation of oxidative stress and neuroinflammation.
	HMOX1	Mangiferin [59]	Protects neurons and glia from the oxidative damage by increasing HMOX1 in AD.
	HMOX1	Luteolin [60]	Increases cells’ survival by preventing apoptosis and oxidative stress.
	* - *	Curcumin [61]	Inhibits the secretion of pro-neuroinflammatory mediators by increasing HMOX.
	-	Curcumin [62,63,64,65]	Protects neurons by ameliorating brain water content, oxidative stress, inflammation, and apoptosis, as well as reversal of depressive-like behaviors.
	*-*	Quercetin, anthocyanins, tea polyphenols, kaempferol, hesperetin, icariin, and various forms of terpenoids [28]	Protect from glutamate neurotoxicity and rescue of impaired cognitive function by increasing antioxidant responses, improving cell viability, and decreasing pro-inflammatory mediators.
	* - *	Curcumin [66]	Improves motor deficits and morphological alterations through antioxidant activity in an in vivo model of quinolinic acid neurotoxicity.
	NRF2 and PERK pathway	Curcumin [67]	Improves motor, sensory, reflex, and balance through inhibition of oxidative stress and apoptotic process.
NFkB/TLR4	NRF2 and NFkB	Curcumin [68]	Improves memory and behavior.
NFkB/STAT3/Ap-1		Luteolin [60]	Reduces neuroinflammation induced by astrocytes.
NFkB/MAPKs	NFkB/MAPK pathways	Curcumin [61]	Inhibits the secretion of pro-neuroinflammatory mediators by increasing Hmox.
NFkB		Ellagic acid [69]	Promotes anti-inflammatory and anti-antioxidant effects in AD and PD.
PKC	PKC activates NRF2	Curcumin [70]	Neurons are stimulated to increase antioxidant gene expression (GST-mu1, NQO1, and Hmox1), as well as p62, resulting in a positive feedback loop.
ERK	ERK modulate NRF2 anti-oxidant signaling	Curcumin [66]	Improve motor deficits and morphological alterations through antioxidant activity.
	AKT2/NRF2 pathways	Kaempferol Ginsenoside rh2 [71]	Upregulation of the antioxidant enzyme thioredoxin linked to antidepressant mechanism.
	AKT/NRF2	Curcumin [72]	Protects neurons and reduces infarct size in in vitro (oxygen and glucose deprivation/reoxygenation) and in vivo (middle cerebral artery occlusion) models of ischemic injury.
NADPH oxidase	Bilirubin, biliverdin	C-phycocyanin (C- PC) [73]	Protective in many neurodegenerative diseases and in COVID-19-induced neurologic damage.
PPARs	Bilirubin [74]	Resveratrol [75,76]	Beneficiary effects on glucose and adipose tissue metabolism.
PGC1a	Bilirubin [77]	Resveratrol, quercetin, curcumin, saponins, epigallocatechin-3-gallate (EGCG) [37]	Regulation of cellular energy metabolism with beneficiary effects on civilization diseases.
CD39	Bilirubin [78] CO [79]	Resveratrol [38] Curcumin [39]	Control of inflammatory processes via purinergic signaling.
FGF21	Bilirubin [80,81]	Coffee phytochemicals (chlorogenic/protocatechuic acid) [82] Cocoa phytochemicals (theobromine/protocatechuic acid) [83]	Energy homeostasis, via FGF21 signaling, a late-acting fed and fasting-state hormone.
mTOR	Bilirubin [10,14] biliverdin [84] CO [85]	Curcumin, quercetin, apigenin [86]	Modulation of nutrient-sensing with impact on intermediary metabolism, aging processes, and overall life span.
SIRT1	Bilirubin [87]	Resveratrol, butein, quercetin [88], astragaloside IV [40]	Control of fat and glucose metabolism, and energy expenditure. Anti-inflammatory and antioxidant. Reducing infarct size in ischemic stroke.
AMPK	Bilirubin [77] biliverdin [89] CO [85]	Resveratrol Berberine Quercetin [90]	Prevention of cardiovascular and metabolic diseases (T2DM), energy homeostasis.
GSK3b	BLVRA/bilirubin [91]	Resveratrol, curcumin, berberine [92]	Modulation of cellular kinase, with >100 known targets affecting lipid and glucose metabolism, and cell proliferation.

## Data Availability

All the data are inside the review.

## References

[1] Gazzin S., Vitek L., Watchko J., Shapiro S.M., Tiribelli C. (2016). A Novel Perspective on the Biology of Bilirubin in Health and Disease. Trends Mol. Med..

[2] Wagner K.-H., Wallner M., Mölzer C., Gazzin S., Bulmer A.C., Tiribelli C., Vitek L. (2015). Looking to the Horizon: The Role of Bilirubin in the Development and Prevention of Age-Related Chronic Diseases. Clin. Sci..

[3] Vitek L., Bellarosa C., Tiribelli C. (2019). Induction of Mild Hyperbilirubinemia: Hype or Real Therapeutic Opportunity?. Clin. Pharmacol. Ther..

[4] Creeden J.F., Gordon D.M., Stec D.E., Hinds T.D. (2020). Bilirubin as a Metabolic Hormone: The Physiological Relevance of Low Levels. Am. J. Physiol.-Endocrinol. Metab..

[5] Jayanti S., Dalla Verde C., Tiribelli C., Gazzin S. (2023). Inflammation, Dopaminergic Brain and Bilirubin. Int. J. Mol. Sci..

[6] Llido J.P., Jayanti S., Tiribelli C., Gazzin S. (2023). Bilirubin and Redox Stress in Age-Related Brain Diseases. Antioxidants.

[7] Ostrow J.D., Vitek L. (2009). Bilirubin Chemistry and Metabolism; Harmful and Protective Aspects. Curr. Pharm. Design.

[8] Ryter S.W., Otterbein L.E. (2004). Carbon Monoxide in Biology and Medicine. BioEssays.

[9] Wegiel B., Otterbein L. (2012). Go Green: The Anti-Inflammatory Effects of Biliverdin Reductase. Front. Pharmacol..

[10] Gazzin S., Masutti F., Vítek L., Tiribelli C. (2016). The Molecular Basis of Jaundice: An Old Symptom Revisited. Liver Int..

[11] Vítek L. (2017). Bilirubin and Atherosclerotic Diseases. Physiol. Res..

[12] Vítek L. (2020). Bilirubin as a Signaling Molecule. Med. Res. Rev..

[13] Vítek L. (2021). The Protective Role of the Heme Catabolic Pathway in Hepatic Disorders. Antioxid. Redox Signal..

[14] Vítek L. (2022). Biliverdin and Bilirubin as Parallel Products of CO Formation. Carbon Monoxide in Drug Discovery.

[15] Ryter S.W., Alam J., Choi A.M.K. (2006). Heme Oxygenase-1/Carbon Monoxide: From Basic Science to Therapeutic Applications. Physiol. Rev..

[16] Wilks A., Knör G., Wu H., Zheng Y., Liu J., Zhang H., Chen H., Buchberger T., Lamparter T., Estes S. (2002). Heme Oxygenase: Evolution, Structure, and Mechanism. Antioxid. Redox Signal..

[17] Maines M.D. (1997). THE HEME OXYGENASE SYSTEM: A Regulator of Second Messenger Gases. Annu. Rev. Pharmacol. Toxicol..

[18] Morse D., Choi A.M.K. (2002). Heme Oxygenase-1. Am. J. Respir. Cell Mol. Biol..

[19] Funes S.C., Rios M., Fernández-Fierro A., Covián C., Bueno S.M., Riedel C.A., Mackern-Oberti J.P., Kalergis A.M. (2020). Naturally Derived Heme-Oxygenase 1 Inducers and Their Therapeutic Application to Immune-Mediated Diseases. Front. Immunol..

[20] Ma Q. (2013). Role of Nrf2 in Oxidative Stress and Toxicity. Annu. Rev. Pharmacol. Toxicol..

[21] Loboda A., Damulewicz M., Pyza E., Jozkowicz A., Dulak J. (2016). Role of Nrf2/HO-1 System in Development, Oxidative Stress Response and Diseases: An Evolutionarily Conserved Mechanism. Cell. Mol. Life Sci..

[22] da Costa R.M., Rodrigues D., Pereira C.A., Silva J.F., Alves J.V., Lobato N.S., Tostes R.C. (2019). Nrf2 as a Potential Mediator of Cardiovascular Risk in Metabolic Diseases. Front. Pharmacol..

[23] Zhang D.D., Chapman E. (2020). The Role of Natural Products in Revealing NRF2 Function. Nat. Prod. Rep..

[24] Qader M., Xu J., Yang Y., Liu Y., Cao S. (2020). Natural Nrf2 Activators from Juices, Wines, Coffee, and Cocoa. Beverages.

[25] Zhang J., Xu H.-X., Zhu J.-Q., Dou Y.-X., Xian Y.-F., Lin Z.-X. (2023). Natural Nrf2 Inhibitors: A Review of Their Potential for Cancer Treatment. Int. J. Biol. Sci..

[26] Robledinos-Antón N., Fernández-Ginés R., Manda G., Cuadrado A. (2019). Activators and Inhibitors of NRF2: A Review of Their Potential for Clinical Development. Oxid. Med. Cell Longev..

[27] Singh S., Nagalakshmi D., Sharma K.K., Ravichandiran V. (2021). Natural Antioxidants for Neuroinflammatory Disorders and Possible Involvement of Nrf2 Pathway: A Review. Heliyon.

[28] Moratilla-Rivera I., Sánchez M., Valdés-González J.A., Gómez-Serranillos M.P. (2023). Natural Products as Modulators of Nrf2 Signaling Pathway in Neuroprotection. Int. J. Mol. Sci..

[29] Puentes-Pardo J.D., Moreno-SanJuan S., Carazo Á., León J. (2020). Heme Oxygenase-1 in Gastrointestinal Tract Health and Disease. Antioxidants.

[30] Song W., Zukor H., Lin S.-H., Hascalovici J., Liberman A., Tavitian A., Mui J., Vali H., Tong X.-K., Bhardwaj S.K. (2012). Schizophrenia-Like Features in Transgenic Mice Overexpressing Human HO-1 in the Astrocytic Compartment. J. Neurosci..

[31] Laskaris L.E., Di Biase M.A., Everall I., Chana G., Christopoulos A., Skafidas E., Cropley V.L., Pantelis C. (2016). Microglial Activation and Progressive Brain Changes in Schizophrenia. Br. J. Pharmacol..

[32] Zhuo C., Tian H., Song X., Jiang D., Chen G., Cai Z., Ping J., Cheng L., Zhou C., Chen C. (2023). Microglia and Cognitive Impairment in Schizophrenia: Translating Scientific Progress into Novel Therapeutic Interventions. Schizophrenia.

[33] Li J., Wang Y., Yuan X., Kang Y., Song X. (2023). New Insight in the Cross-Talk between Microglia and Schizophrenia: From the Perspective of Neurodevelopment. Front. Psychiatry.

[34] Fleiss B., Van Steenwinckel J., Bokobza C., Shearer I.K., Ross-Munro E., Gressens P. (2021). Microglia-Mediated Neurodegeneration in Perinatal Brain Injuries. Biomolecules.

[35] Schipper H.M., Song W., Tavitian A., Cressatti M. (2019). The Sinister Face of Heme Oxygenase-1 in Brain Aging and Disease. Progress. Neurobiol..

[36] Bereczki D., Balla J., Bereczki D. (2018). Heme Oxygenase-1: Clinical Relevance in Ischemic Stroke. Curr. Pharm. Des..

[37] Suntar I., Sureda A., Belwal T., Sanches Silva A., Vacca R.A., Tewari D., Sobarzo-Sánchez E., Nabavi S.F., Shirooie S., Dehpour A.R. (2020). Natural Products, PGC-1 α, and Duchenne Muscular Dystrophy. Acta Pharm. Sin. B.

[38] Bottari N.B., Reichert K.P., Fracasso M., Dutra A., Assmann C.E., Ulrich H., Schetinger M.R.C., Morsch V.M., Da Silva A.S. (2020). Neuroprotective Role of Resveratrol Mediated by Purinergic Signalling in Cerebral Cortex of Mice Infected by Toxoplasma Gondii. Parasitol. Res..

[39] Costa P., Gonçalves J., Baldissarelli J., Mann T., Abdalla F., Fiorenza A., Rosa M., Carvalho F., Gutierres J., Andrade C. (2015). Curcumin Attenuates Memory Deficits and the Impairment of Cholinergic and Purinergic Signaling in Rats Chronically Exposed to Cadmium. Environ. Toxicol..

[40] Zhang Y., Zhang Y., Jin X., Zhou X., Dong X., Yu W., Gao W. (2019). The Role of Astragaloside IV against Cerebral Ischemia/Reperfusion Injury: Suppression of Apoptosis via Promotion of P62-LC3-Autophagy. Molecules.

[41] Nitti M., Piras S., Brondolo L., Marinari U.M., Pronzato M.A., Furfaro A.L. (2018). Heme Oxygenase 1 in the Nervous System: Does It Favor Neuronal Cell Survival or Induce Neurodegeneration?. Int. J. Mol. Sci..

[42] Dwyer B.E., Nishimura R.N., Lu S.Y. (1995). Differential Expression of Heme Oxygenase-1 in Cultured Cortical Neurons and Astrocytes Determined by the Aid of a New Heme Oxygenase Antibody. Response to Oxidative Stress. Brain Res. Mol. Brain Res..

[43] Cheng Q., Shen Y., Cheng Z., Shao Q., Wang C., Sun H., Zhang Q. (2019). Achyranthes Bidentata Polypeptide k Suppresses Neuroinflammation in BV2 Microglia through Nrf2-Dependent Mechanism. Ann. Transl. Med..

[44] Jayanti S., Vítek L., Tiribelli C., Gazzin S. (2020). The Role of Bilirubin and the Other “Yellow Players” in Neurodegenerative Diseases. Antioxidants.

[45] Sahebnasagh A., Eghbali S., Saghafi F., Sureda A., Avan R. (2022). Neurohormetic Phytochemicals in the Pathogenesis of Neurodegenerative Diseases. Immun. Ageing.

[46] Marino A., Battaglini M., Moles N., Ciofani G. (2022). Natural Antioxidant Compounds as Potential Pharmaceutical Tools against Neurodegenerative Diseases. ACS Omega.

[47] Huang J.-Y., Yuan Y.-H., Yan J.-Q., Wang Y.-N., Chu S.-F., Zhu C.-G., Guo Q.-L., Shi J.-G., Chen N.-H. (2016). 20C, a Bibenzyl Compound Isolated from Gastrodia Elata, Protects PC12 Cells against Rotenone-Induced Apoptosis via Activation of the Nrf2/ARE/HO-1 Signaling Pathway. Acta Pharmacol. Sin..

[48] Cordaro M., Modafferi S., D’Amico R., Fusco R., Genovese T., Peritore A.F., Gugliandolo E., Crupi R., Interdonato L., Di Paola D. (2022). Natural Compounds Such as *Hericium erinaceus* and *Coriolus versicolor* Modulate Neuroinflammation, Oxidative Stress and Lipoxin A4 Expression in Rotenone-Induced Parkinson’s Disease in Mice. Biomedicines.

[49] Duan C., Wang H., Jiao D., Geng Y., Wu Q., Yan H., Li C. (2022). Curcumin Restrains Oxidative Stress of After Intracerebral Hemorrhage in Rat by Activating the Nrf2/HO-1 Pathway. Front. Pharmacol..

[50] Trock B., Lanza E., Greenwald P. (1990). Dietary Fiber, Vegetables, and Colon Cancer: Critical Review and Meta-Analyses of the Epidemiologic Evidence. J. Natl. Cancer Inst..

[51] Qaisiya M., Coda Zabetta C.D., Bellarosa C., Tiribelli C. (2014). Bilirubin Mediated Oxidative Stress Involves Antioxidant Response Activation via Nrf2 Pathway. Cell. Signal..

[52] Kwon S.-H., Lee S.R., Park Y.J., Ra M., Lee Y., Pang C., Kim K.H. (2019). Suppression of 6-Hydroxydopamine-Induced Oxidative Stress by Hyperoside via Activation of Nrf2/HO-1 Signaling in Dopaminergic Neurons. Int. J. Mol. Sci..

[53] Zhang C., Li C., Chen S., Li Z., Jia X., Wang K., Bao J., Liang Y., Wang X., Chen M. (2017). Berberine Protects against 6-OHDA-Induced Neurotoxicity in PC12 Cells and Zebrafish through Hormetic Mechanisms Involving PI3K/AKT/Bcl-2 and Nrf2/HO-1 Pathways. Redox Biol..

[54] Li F., Wang X., Zhang Z., Gao P., Zhang X. (2019). Breviscapine Provides a Neuroprotective Effect after Traumatic Brain Injury by Modulating the Nrf2 Signaling Pathway. J. Cell Biochem..

[55] Lee D.-S., Cha B.-Y., Woo J.-T., Kim Y.-C., Jang J.-H. (2015). Acerogenin A from *Acer nikoense* Maxim Prevents Oxidative Stress-Induced Neuronal Cell Death through Nrf2-Mediated Heme Oxygenase-1 Expression in Mouse Hippocampal HT22 Cell Line. Molecules.

[56] Gu D.-M., Lu P.-H., Zhang K., Wang X., Sun M., Chen G.-Q., Wang Q. (2015). EGFR Mediates Astragaloside IV-Induced Nrf2 Activation to Protect Cortical Neurons against in vitro Ischemia/Reperfusion Damages. Biochem. Biophys. Res. Commun..

[57] Li C., Yang F., Liu F., Li D., Yang T. (2018). NRF2/HO-1 Activation via ERK Pathway Involved in the Anti-Neuroinflammatory Effect of Astragaloside IV in LPS Induced Microglial Cells. Neurosci. Lett..

[58] Baluchnejadmojarad T., Kiasalari Z., Afshin-Majd S., Ghasemi Z., Roghani M. (2017). S-Allyl Cysteine Ameliorates Cognitive Deficits in Streptozotocin-Diabetic Rats via Suppression of Oxidative Stress, Inflammation, and Acetylcholinesterase. Eur. J. Pharmacol..

[59] Feng S.-T., Wang Z.-Z., Yuan Y.-H., Sun H.-M., Chen N.-H., Zhang Y. (2019). Mangiferin: A Multipotent Natural Product Preventing Neurodegeneration in Alzheimer’s and Parkinson’s Disease Models. Pharmacol. Res..

[60] Siddique Y.H. (2021). Role of Luteolin in Overcoming Parkinson’s Disease. Biofactors.

[61] Jin M., Park S.Y., Shen Q., Lai Y., Ou X., Mao Z., Lin D., Yu Y., Zhang W. (2018). Anti-Neuroinflammatory Effect of Curcumin on Pam3CSK4-Stimulated Microglial Cells. Int. J. Mol. Med..

[62] Dai W., Wang H., Fang J., Zhu Y., Zhou J., Wang X., Zhou Y., Zhou M. (2018). Curcumin Provides Neuroprotection in Model of Traumatic Brain Injury via the Nrf2-ARE Signaling Pathway. Brain Res. Bull..

[63] Dong W., Yang B., Wang L., Li B., Guo X., Zhang M., Jiang Z., Fu J., Pi J., Guan D. (2018). Curcumin Plays Neuroprotective Roles against Traumatic Brain Injury Partly via Nrf2 Signaling. Toxicol. Appl. Pharmacol..

[64] Yang B., Yin C., Zhou Y., Wang Q., Jiang Y., Bai Y., Qian H., Xing G., Wang S., Li F. (2019). Curcumin Protects against Methylmercury-Induced Cytotoxicity in Primary Rat Astrocytes by Activating the Nrf2/ARE Pathway Independently of PKCδ. Toxicology.

[65] Liao D., Lv C., Cao L., Yao D., Wu Y., Long M., Liu N., Jiang P. (2020). Curcumin Attenuates Chronic Unpredictable Mild Stress-Induced Depressive-Like Behaviors via Restoring Changes in Oxidative Stress and the Activation of Nrf2 Signaling Pathway in Rats. Oxidative Med. Cell. Longev..

[66] Santana-Martínez R.A., Silva-Islas C.A., Fernández-Orihuela Y.Y., Barrera-Oviedo D., Pedraza-Chaverri J., Hernández-Pando R., Maldonado P.D. (2019). The Therapeutic Effect of Curcumin in Quinolinic Acid-Induced Neurotoxicity in Rats Is Associated with BDNF, ERK1/2, Nrf2, and Antioxidant Enzymes. Antioxidants.

[67] Huang T., Zhao J., Guo D., Pang H., Zhao Y., Song J. (2018). Curcumin Mitigates Axonal Injury and Neuronal Cell Apoptosis through the PERK/Nrf2 Signaling Pathway Following Diffuse Axonal Injury. NeuroReport.

[68] Ikram M., Saeed K., Khan A., Muhammad T., Khan M.S., Jo M.G., Rehman S.U., Kim M.O. (2019). Natural Dietary Supplementation of Curcumin Protects Mice Brains against Ethanol-Induced Oxidative Stress-Mediated Neurodegeneration and Memory Impairment via Nrf2/TLR4/RAGE Signaling. Nutrients.

[69] Zhu H., Yan Y., Jiang Y., Meng X. (2022). Ellagic Acid and Its Anti-Aging Effects on Central Nervous System. Int. J. Mol. Sci..

[70] Park J.-Y., Sohn H.-Y., Koh Y.H., Jo C. (2021). Curcumin Activates Nrf2 through PKCδ-Mediated P62 Phosphorylation at Ser351. Sci. Rep..

[71] Li X., Wang Y., Wang C., Jing R., Mu L., Liu P., Hu Y. (2022). Antidepressant Mechanism of Kaixinsan and Its Active Compounds Based on Upregulation of Antioxidant Thioredoxin. Evid. -Based Complement. Altern. Med..

[72] Wu J., Li Q., Wang X., Yu S., Li L., Wu X., Chen Y., Zhao J., Zhao Y. (2013). Neuroprotection by Curcumin in Ischemic Brain Injury Involves the Akt/Nrf2 Pathway. PLoS ONE.

[73] Pentón-Rol G., Marín-Prida J., McCarty M.F. (2021). C-Phycocyanin-Derived Phycocyanobilin as a Potential Nutraceutical Approach for Major Neurodegenerative Disorders and COVID-19-Induced Damage to the Nervous System. Curr. Neuropharmacol..

[74] Vitek L., Hinds T.D., Stec D.E., Tiribelli C. (2023). The Physiology of Bilirubin: Health and Disease Equilibrium. Trends Mol. Med..

[75] Wang L., Waltenberger B., Pferschy-Wenzig E.-M., Blunder M., Liu X., Malainer C., Blazevic T., Schwaiger S., Rollinger J.M., Heiss E.H. (2014). Natural Product Agonists of Peroxisome Proliferator-Activated Receptor Gamma (PPARγ): A Review. Biochem. Pharmacol..

[76] Rigano D., Sirignano C., Taglialatela-Scafati O. (2017). The Potential of Natural Products for Targeting PPARα. Acta Pharm. Sin. B.

[77] Mölzer C., Wallner M., Kern C., Tosevska A., Schwarz U., Zadnikar R., Doberer D., Marculescu R., Wagner K.-H. (2016). Features of an Altered AMPK Metabolic Pathway in Gilbert’s Syndrome, and Its Role in Metabolic Health. Sci. Rep..

[78] Longhi M.S., Vuerich M., Kalbasi A., Kenison J.E., Yeste A., Csizmadia E., Vaughn B., Feldbrugge L., Mitsuhashi S., Wegiel B. (2017). Bilirubin Suppresses Th17 Immunity in Colitis by Upregulating CD39. JCI Insight.

[79] Correa-Costa M., Gallo D., Csizmadia E., Gomperts E., Lieberum J.-L., Hauser C.J., Ji X., Wang B., Câmara N.O.S., Robson S.C. (2018). Carbon Monoxide Protects the Kidney through the Central Circadian Clock and CD39. Proc. Natl. Acad. Sci. USA.

[80] McCarty M.F. (2017). Practical Prospects for Boosting Hepatic Production of the “pro-Longevity” Hormone FGF21. Horm. Mol. Biol. Clin. Investig..

[81] Hinds T.D., Stec D.E. (2018). Bilirubin, a Cardiometabolic Signaling Molecule. Hypertension.

[82] Rebollo-Hernanz M., Aguilera Y., Martín-Cabrejas M.A., Gonzalez de Mejia E. (2022). Activating Effects of the Bioactive Compounds From Coffee By-Products on FGF21 Signaling Modulate Hepatic Mitochondrial Bioenergetics and Energy Metabolism in vitro. Front. Nutr..

[83] Rebollo-Hernanz M., Aguilera Y., Martin-Cabrejas M.A., Gonzalez de Mejia E. (2022). Phytochemicals from the Cocoa Shell Modulate Mitochondrial Function, Lipid and Glucose Metabolism in Hepatocytes via Activation of FGF21/ERK, AKT, and mTOR Pathways. Antioxidants.

[84] Lanzillotta C., Zuliani I., Vasavda C., Snyder S.H., Paul B.D., Perluigi M., Di Domenico F.D., Barone E. (2020). BVR-A Deficiency Leads to Autophagy Impairment through the Dysregulation of AMPK/mTOR Axis in the Brain-Implications for Neurodegeneration. Antioxidants.

[85] Kim H.J., Joe Y., Kim S.-K., Park S.-U., Park J., Chen Y., Kim J., Ryu J., Cho G.J., Surh Y.-J. (2017). Carbon Monoxide Protects against Hepatic Steatosis in Mice by Inducing Sestrin-2 via the PERK-eIF2α-ATF4 Pathway. Free Radic. Biol. Med..

[86] Huang S. (2013). Inhibition of PI3K/Akt/mTOR Signaling by Natural Products. Anticancer. Agents Med. Chem..

[87] Vakili O., Borji M., Saffari-Chaleshtori J., Shafiee S.M. (2023). Ameliorative Effects of Bilirubin on Cell Culture Model of Non-Alcoholic Fatty Liver Disease. Mol. Biol. Rep..

[88] Alcaín F.J., Villalba J.M. (2009). Sirtuin Activators. Expert. Opin. Ther. Pat..

[89] Zhang Z., Amorosa L.F., Petrova A., Coyle S., Macor M., Nair M., Lee L.Y., Haimovich B. (2019). TLR4 Counteracts BVRA Signaling in Human Leukocytes via Differential Regulation of AMPK, mTORC1 and mTORC2. Sci. Rep..

[90] Heidary Moghaddam R., Samimi Z., Asgary S., Mohammadi P., Hozeifi S., Hoseinzadeh-Chahkandak F., Xu S., Farzaei M.H. (2021). Natural AMPK Activators in Cardiovascular Disease Prevention. Front. Pharmacol..

[91] Hinds T.D., Burns K.A., Hosick P.A., McBeth L., Nestor-Kalinoski A., Drummond H.A., AlAmodi A.A., Hankins M.W., Vanden Heuvel J.P., Stec D.E. (2016). Biliverdin Reductase A Attenuates Hepatic Steatosis by Inhibition of Glycogen Synthase Kinase (GSK) 3β Phosphorylation of Serine 73 of Peroxisome Proliferator-Activated Receptor (PPAR) α. J. Biol. Chem..

[92] Duda P., Akula S.M., Abrams S.L., Steelman L.S., Martelli A.M., Cocco L., Ratti S., Candido S., Libra M., Montalto G. (2020). Targeting GSK3 and Associated Signaling Pathways Involved in Cancer. Cells.

[93] Song X., Long D. (2020). Nrf2 and Ferroptosis: A New Research Direction for Neurodegenerative Diseases. Front. Neurosci..

[94] Gozzelino R. (2016). The Pathophysiology of Heme in the Brain. Curr. Alzheimer Res..

[95] Schipper H.M. (2004). Brain Iron Deposition and the Free Radical-Mitochondrial Theory of Ageing. Ageing Res. Rev..

[96] Ozen M., Kitase Y., Vasan V., Burkhardt C., Ramachandra S., Robinson S., Jantzie L.L. (2021). Chorioamnionitis Precipitates Perinatal Alterations of Heme-Oxygenase-1 (HO-1) Homeostasis in the Developing Rat Brain. Int. J. Mol. Sci..

[97] Kram H., Prokop G., Haller B., Gempt J., Wu Y., Schmidt-Graf F., Schlegel J., Conrad M., Liesche-Starnecker F. (2022). Glioblastoma Relapses Show Increased Markers of Vulnerability to Ferroptosis. Front. Oncol..

[98] Hara E., Takahashi K., Tominaga T., Kumabe T., Kayama T., Suzuki H., Fujita H., Yoshimoto T., Shirato K., Shibahara S. (1996). Expression of Heme Oxygenase and Inducible Nitric Oxide Synthase mRNA in Human Brain Tumors. Biochem. Biophys. Res. Commun..

[99] Vandenbark A.A., Offner H., Matejuk S., Matejuk A. (2021). Microglia and Astrocyte Involvement in Neurodegeneration and Brain Cancer. J. Neuroinflammation.

[100] Maas S.L.N., Abels E.R., Van De Haar L.L., Zhang X., Morsett L., Sil S., Guedes J., Sen P., Prabhakar S., Hickman S.E. (2020). Glioblastoma Hijacks Microglial Gene Expression to Support Tumor Growth. J. Neuroinflammation.

[101] Catalano M., Serpe C., Limatola C. (2022). Microglial Extracellular Vesicles as Modulators of Brain Microenvironment in Glioma. Int. J. Mol. Sci..

[102] Lanza M., Casili G., Campolo M., Paterniti I., Colarossi C., Mare M., Giuffrida R., Caffo M., Esposito E., Cuzzocrea S. (2021). Immunomodulatory Effect of Microglia-Released Cytokines in Gliomas. Brain Sci..

[103] Haghshenas M.R., Saffarian A., Khademolhosseini A., Dehghanian A., Ghaderi A., Sotoodeh Jahromi A. (2022). Simultaneous Increase in Serum Levels of IL-37 and IL-18 Binding Protein In Low-Grade and High-Grade Brain Tumors. Asian Pac. J. Cancer Prev..

[104] Stec D.E., John K., Trabbic C.J., Luniwal A., Hankins M.W., Baum J., Hinds T.D. (2016). Bilirubin Binding to PPARα Inhibits Lipid Accumulation. PLoS ONE.

[105] Pepino M.Y., Kuda O., Samovski D., Abumrad N.A. (2014). Structure-Function of CD36 and Importance of Fatty Acid Signal Transduction in Fat Metabolism. Annu. Rev. Nutr..

[106] Lee J.H., Wada T., Febbraio M., He J., Matsubara T., Lee M.J., Gonzalez F.J., Xie W. (2010). A Novel Role for the Dioxin Receptor in Fatty Acid Metabolism and Hepatic Steatosis. Gastroenterology.

[107] Phelan D., Winter G.M., Rogers W.J., Lam J.C., Denison M.S. (1998). Activation of the Ah Receptor Signal Transduction Pathway by Bilirubin and Biliverdin. Arch. Biochem. Biophys..

[108] Gordon D.M., Blomquist T.M., Miruzzi S.A., McCullumsmith R., Stec D.E., Hinds T.D. (2019). RNA Sequencing in Human HepG2 Hepatocytes Reveals PPAR-α Mediates Transcriptome Responsiveness of Bilirubin. Physiol. Genom..

[109] Nakao A., Murase N., Ho C., Toyokawa H., Billiar T.R., Kanno S. (2005). Biliverdin Administration Prevents the Formation of Intimal Hyperplasia Induced by Vascular Injury. Circulation.

[110] Deguchi K., Hayashi T., Nagotani S., Sehara Y., Zhang H., Tsuchiya A., Ohta Y., Tomiyama K., Morimoto N., Miyazaki M. (2008). Reduction of Cerebral Infarction in Rats by Biliverdin Associated with Amelioration of Oxidative Stress. Brain Res..

[111] Zou Z.-Y., Liu J., Chang C., Li J.-J., Luo J., Jin Y., Ma Z., Wang T.-H., Shao J.-L. (2019). Biliverdin Administration Regulates the microRNA-mRNA Expressional Network Associated with Neuroprotection in Cerebral Ischemia Reperfusion Injury in Rats. Int. J. Mol. Med..

[112] Triani F., Tramutola A., Di Domenico F., Sharma N., Butterfield D.A., Head E., Perluigi M., Barone E. (2018). Biliverdin Reductase-A Impairment Links Brain Insulin Resistance with Increased Aβ Production in an Animal Model of Aging: Implications for Alzheimer Disease. Biochim. Et Biophys. Acta (BBA)—Mol. Basis Dis..

[113] Barone E., Mancuso C., Di Domenico F., Sultana R., Murphy M.P., Head E., Butterfield D.A. (2012). Biliverdin Reductase-A: A Novel Drug Target for Atorvastatin in a Dog Pre-Clinical Model of Alzheimer Disease. J. Neurochem..

[114] Gibbs P.E.M., Maines M.D. (2007). Biliverdin Inhibits Activation of NF-κB: Reversal of Inhibition by Human Biliverdin Reductase. Int. J. Cancer.

[115] Atukeren P., Oner S., Baran O., Kemerdere R., Eren B., Cakatay U., Tanriverdi T. (2017). Oxidant and Anti-Oxidant Status in Common Brain Tumors: Correlation to TP53 and Human Biliverdin Reductase. Clin. Neurol. Neurosurg..

[116] Zaghloul N., Kurepa D., Bader M.Y., Nagy N., Ahmed M.N. (2020). Prophylactic Inhibition of NF-κB Expression in Microglia Leads to Attenuation of Hypoxic Ischemic Injury of the Immature Brain. J. Neuroinflammation.

[117] Costa-De-Santana B.J.R., Manhães-De-Castro R., Gouveia H.J.C.B., Silva E.R., Araújo M.A.d.S., Lacerda D.C., Guzmán-Quevedo O., Torner L., Toscano A.E. (2023). Motor Deficits Are Associated with Increased Glial Cell Activation in the Hypothalamus and Cerebellum of Young Rats Subjected to Cerebral Palsy. Brain Res..

[118] Mallard C., Davidson J.O., Tan S., Green C.R., Bennet L., Robertson N.J., Gunn A.J. (2014). Astrocytes and Microglia in Acute Cerebral Injury Underlying Cerebral Palsy Associated with Preterm Birth. Pediatr. Res..

[119] Hu C., Li H., Li J., Luo X., Hao Y. (2022). Microglia: Synaptic Modulator in Autism Spectrum Disorder. Front. Psychiatry.

[120] Brégère C., Schwendele B., Radanovic B., Guzman R. (2022). Microglia and Stem-Cell Mediated Neuroprotection after Neonatal Hypoxia-Ischemia. Stem Cell Rev. Rep..

[121] Zhang Y., Xie Y., Cheng Z., Zhang Y., Wang W., Guo B., Wu S. (2022). Mechanism of Action and Therapeutic Targeting of Microglia in Autism Spectrum Disorder. Adv. Neurol..

[122] Zhang F., Nance E., Alnasser Y., Kannan R., Kannan S. (2016). Microglial Migration and Interactions with Dendrimer Nanoparticles Are Altered in the Presence of Neuroinflammation. J. Neuroinflammation.

[123] Davoli-Ferreira M., Thomson C.A., McCoy K.D. (2021). Microbiota and Microglia Interactions in ASD. Front. Immunol..

[124] Koyama R., Ikegaya Y. (2015). Microglia in the Pathogenesis of Autism Spectrum Disorders. Neurosci. Res..

[125] Tsilioni I., Patel A.B., Pantazopoulos H., Berretta S., Conti P., Leeman S.E., Theoharides T.C. (2019). IL-37 Is Increased in Brains of Children with Autism Spectrum Disorder and Inhibits Human Microglia Stimulated by Neurotensin. Proc. Natl. Acad. Sci. USA.

[126] Vítek L., Tiribelli C. (2023). Gilbert’s Syndrome Revisited. J. Hepatol..

[127] Sugatani J., Mizushima K., Osabe M., Yamakawa K., Kakizaki S., Takagi H., Mori M., Ikari A., Miwa M. (2008). Transcriptional Regulation of Human UGT1A1 Gene Expression through Distal and Proximal Promoter Motifs: Implication of Defects in the UGT1A1 Gene Promoter. Naunyn Schmiedebergs Arch. Pharmacol..

[128] Bock K.W., Köhle C. (2010). Contributions of the Ah Receptor to Bilirubin Homeostasis and Its Antioxidative and Atheroprotective Functions. Biol. Chem..

[129] Xiao L., Zhang Z., Luo X. (2014). Roles of Xenobiotic Receptors in Vascular Pathophysiology. Circ. J..

[130] Jayanti S., Moretti R., Tiribelli C., Gazzin S. (2022). Bilirubin Prevents the TH+ Dopaminergic Neuron Loss in a Parkinson’s Disease Model by Acting on TNF-α. Int. J. Mol. Sci..

[131] Hernandez J.P., Mota L.C., Baldwin W.S. (2009). Activation of CAR and PXR by Dietary, Environmental and Occupational Chemicals Alters Drug Metabolism, Intermediary Metabolism, and Cell Proliferation. Curr. Pharmacogenomics Person. Med..

[132] Busbee P.B., Rouse M., Nagarkatti M., Nagarkatti P.S. (2013). Use of Natural AhR Ligands as Potential Therapeutic Modalities against Inflammatory Disorders. Nutr. Rev..

[133] Hong F., Pan S., Guo Y., Xu P., Zhai Y. (2019). PPARs as Nuclear Receptors for Nutrient and Energy Metabolism. Molecules.

[134] Bragt M.C.E., Popeijus H.E. (2008). Peroxisome Proliferator-Activated Receptors and the Metabolic Syndrome. Physiol. Behav..

[135] Duszka K., Gregor A., Guillou H., König J., Wahli W. (2020). Peroxisome Proliferator-Activated Receptors and Caloric Restriction—Common Pathways Affecting Metabolism, Health, and Longevity. Cells.

[136] Potthoff M.J., Kliewer S.A., Mangelsdorf D.J. (2012). Endocrine Fibroblast Growth Factors 15/19 and 21: From Feast to Famine. Genes. Dev..

[137] Liu J., Dong H., Zhang Y., Cao M., Song L., Pan Q., Bulmer A., Adams D.B., Dong X., Wang H. (2015). Bilirubin Increases Insulin Sensitivity by Regulating Cholesterol Metabolism, Adipokines and PPARγ Levels. Sci. Rep..

[138] Dong H., Huang H., Yun X., Kim D., Yue Y., Wu H., Sutter A., Chavin K.D., Otterbein L.E., Adams D.B. (2014). Bilirubin Increases Insulin Sensitivity in Leptin-Receptor Deficient and Diet-Induced Obese Mice through Suppression of ER Stress and Chronic Inflammation. Endocrinology.

[139] Zhang F., Guan W., Fu Z., Zhou L., Guo W., Ma Y., Gong Y., Jiang W., Liang H., Zhou H. (2020). Relationship between Serum Indirect Bilirubin Level and Insulin Sensitivity: Results from Two Independent Cohorts of Obese Patients with Impaired Glucose Regulation and Type 2 Diabetes Mellitus in China. Int. J. Endocrinol..

[140] Lin L.-Y., Kuo H.-K., Hwang J.-J., Lai L.-P., Chiang F.-T., Tseng C.-D., Lin J.-L. (2009). Serum Bilirubin Is Inversely Associated with Insulin Resistance and Metabolic Syndrome among Children and Adolescents. Atherosclerosis.

[141] Shao X., Wang M., Wei X., Deng S., Fu N., Peng Q., Jiang Y., Ye L., Xie J., Lin Y. (2016). Peroxisome Proliferator-Activated Receptor-γ: Master Regulator of Adipogenesis and Obesity. Curr. Stem Cell Res. Ther..

[142] Wang L., Yin Y., Hou G., Kang J., Wang Q. (2018). Peroxisome Proliferator-Activated Receptor (PPARγ) Plays a Protective Role in Cigarette Smoking-Induced Inflammation via AMP-Activated Protein Kinase (AMPK) Signaling. Med. Sci. Monit..

[143] He G., Sung Y.M., Digiovanni J., Fischer S.M. (2006). Thiazolidinediones Inhibit Insulin-like Growth Factor-i-Induced Activation of p70S6 Kinase and Suppress Insulin-like Growth Factor-I Tumor-Promoting Activity. Cancer Res..

[144] Hinds T.D., Hosick P.A., Chen S., Tukey R.H., Hankins M.W., Nestor-Kalinoski A., Stec D.E., Creeden J.F., Gordon D.M., Hipp J.A. (2017). Mice with Hyperbilirubinemia Due to Gilbert’s Syndrome Polymorphism Are Resistant to Hepatic Steatosis by Decreased Serine 73 Phosphorylation of PPARα. Am. J. Physiol. Endocrinol. Metab..

[145] Viollet B., Guigas B., Leclerc J., Hébrard S., Lantier L., Mounier R., Andreelli F., Foretz M. (2009). AMP-Activated Protein Kinase in the Regulation of Hepatic Energy Metabolism: From Physiology to Therapeutic Perspectives. Acta Physiol..

[146] Lin S.-C., Hardie D.G. (2018). AMPK: Sensing Glucose as Well as Cellular Energy Status. Cell Metab..

[147] Stallone G., Infante B., Prisciandaro C., Grandaliano G. (2019). mTOR and Aging: An Old Fashioned Dress. Int. J. Mol. Sci..

[148] González A., Hall M.N., Lin S.-C., Hardie D.G. (2020). AMPK and TOR: The Yin and Yang of Cellular Nutrient Sensing and Growth Control. Cell Metab..

[149] Zelenka J., Dvořák A., Alán L., Zadinová M., Haluzík M., Vítek L. (2016). Hyperbilirubinemia Protects against Aging-Associated Inflammation and Metabolic Deterioration. Oxid. Med. Cell Longev..

[150] Liang H., Ward W.F., Shute R.J., Heesch M.W., Zak R.B., Kreiling J.L., Slivka D.R., Sun S., Li H., Chen J. (2006). PGC-1alpha: A Key Regulator of Energy Metabolism. Adv. Physiol. Educ..

[151] Jiang W. (2008). Sirtuins: Novel Targets for Metabolic Disease in Drug Development. Biochem. Biophys. Res. Commun..

[152] Cantó C., Auwerx J. (2009). PGC-1alpha, SIRT1 and AMPK, an Energy Sensing Network That Controls Energy Expenditure. Curr. Opin. Lipidol..

[153] Shi Y.-H., Zhang X.-L., Ying P.-J., Wu Z.-Q., Lin L.-L., Chen W., Zheng G.-Q., Zhu W.-Z. (2021). Neuroprotective Effect of Astragaloside IV on Cerebral Ischemia/Reperfusion Injury Rats Through Sirt1/Mapt Pathway. Front. Pharmacol..

[154] Antonioli L., Pacher P., Vizi E.S., Haskó G. (2013). CD39 and CD73 in Immunity and Inflammation. Trends Mol. Med..

[155] Lee G.R., Shaefi S., Otterbein L.E. (2019). HO-1 and CD39: It Takes Two to Protect the Realm. Front. Immunol..

[156] Enjyoji K., Kotani K., Thukral C., Blumel B., Sun X., Wu Y., Imai M., Friedman D., Csizmadia E., Bleibel W. (2008). Deletion of Cd39/Entpd1 Results in Hepatic Insulin Resistance. Diabetes.

[157] da Silva C.G., Jarzyna R., Specht A., Kaczmarek E. (2006). Extracellular Nucleotides and Adenosine Independently Activate AMP-Activated Protein Kinase in Endothelial Cells. Circ. Res..

[158] Wang P., Jia J., Zhang D. (2020). Purinergic Signalling in Liver Diseases: Pathological Functions and Therapeutic Opportunities. JHEP Rep..

[159] Wang S., Gao S., Zhou D., Qian X., Luan J., Lv X. (2021). The Role of the CD39-CD73-Adenosine Pathway in Liver Disease. J. Cell Physiol..

[160] Andersson C., Weeke P., Fosbøl E.L., Brendorp B., Køber L., Coutinho W., Sharma A.M., Van Gaal L., Finer N., James W.P.T. (2009). Acute Effect of Weight Loss on Levels of Total Bilirubin in Obese, Cardiovascular High-Risk Patients: An Analysis from the Lead-in Period of the Sibutramine Cardiovascular Outcome Trial. Metabolism.

[161] Chen L., Duan Y., Wei H., Ning H., Bi C., Zhao Y., Qin Y., Li Y. (2019). Acetyl-CoA Carboxylase (ACC) as a Therapeutic Target for Metabolic Syndrome and Recent Developments in ACC1/2 Inhibitors. Expert. Opin. Investig. Drugs.

[162] Ahmad F., Woodgett J.R. (2020). Emerging Roles of GSK-3α in Pathophysiology: Emphasis on Cardio-Metabolic Disorders. Biochim. Biophys. Acta Mol. Cell Res..

[163] Beurel E., Grieco S.F., Jope R.S. (2015). Glycogen Synthase Kinase-3 (GSK3): Regulation, Actions, and Diseases. Pharmacol. Ther..

[164] Bösch F., Thomas M., Kogler P., Oberhuber R., Sucher R., Aigner F., Semsroth S., Wiedemann D., Yamashita K., Troppmair J. (2014). Bilirubin Rinse of the Graft Ameliorates Ischemia Reperfusion Injury in Heart Transplantation. Transpl. Int..

[165] Liu Z., Cao W. (2009). P38 Mitogen-Activated Protein Kinase: A Critical Node Linking Insulin Resistance and Cardiovascular Diseases in Type 2 Diabetes Mellitus. Endocr. Metab. Immune Disord. Drug Targets.

[166] Šuk J., Jašprová J., Biedermann D., Petrásková L., Valentová K., Křen V., Muchová L., Vítek L. (2019). Isolated Silymarin Flavonoids Increase Systemic and Hepatic Bilirubin Concentrations and Lower Lipoperoxidation in Mice. Oxidative Med. Cell. Longev..

[167] Flaig T.W., Gustafson D.L., Su L.-J., Zirrolli J.A., Crighton F., Harrison G.S., Pierson A.S., Agarwal R., Glodé L.M. (2007). A Phase I and Pharmacokinetic Study of Silybin-Phytosome in Prostate Cancer Patients. Investig. New Drugs.

[168] Mariño Z., Crespo G., D’Amato M., Brambilla N., Giacovelli G., Rovati L., Costa J., Navasa M., Forns X. (2013). Intravenous Silibinin Monotherapy Shows Significant Antiviral Activity in HCV-Infected Patients in the Peri-Transplantation Period. J. Hepatol..

[169] Maher P. (2019). The Potential of Flavonoids for the Treatment of Neurodegenerative Diseases. Int. J. Mol. Sci..

[170] Wang T.H., Wang S.Y., Wang X.D., Jiang H.Q., Yang Y.Q., Wang Y., Cheng J.L., Zhang C.T., Liang W.W., Feng H.L. (2018). Fisetin Exerts Antioxidant and Neuroprotective Effects in Multiple Mutant hSOD1 Models of Amyotrophic Lateral Sclerosis by Activating ERK. Neuroscience.

[171] Li L., Li W.-J., Zheng X.-R., Liu Q.-L., Du Q., Lai Y.-J., Liu S.-Q. (2022). Eriodictyol Ameliorates Cognitive Dysfunction in APP/PS1 Mice by Inhibiting Ferroptosis via Vitamin D Receptor-Mediated Nrf2 Activation. Mol. Med..

[172] Mhillaj E., Tarozzi A., Pruccoli L., Cuomo V., Trabace L., Mancuso C. (2019). Curcumin and Heme Oxygenase: Neuroprotection and Beyond. Int. J. Mol. Sci..

[173] Ahmadi M., Agah E., Nafissi S., Jaafari M.R., Harirchian M.H., Sarraf P., Faghihi-Kashani S., Hosseini S.J., Ghoreishi A., Aghamollaii V. (2018). Safety and Efficacy of Nanocurcumin as Add-on Therapy to Riluzole in Patients with Amyotrophic Lateral Sclerosis: A Pilot Randomized Clinical Trial. Neurotherapeutics.

[174] Valverde-Salazar V., Ruiz-Gabarre D., García-Escudero V. (2023). Alzheimer’s Disease and Green Tea: Epigallocatechin-3-Gallate as a Modulator of Inflammation and Oxidative Stress. Antioxidants.

[175] Mohi-Ud-Din R., Mir R.H., Shah A.J., Sabreen S., Wani T.U., Masoodi M.H., Akkol E.K., Bhat Z.A., Khan H. (2022). Plant-Derived Natural Compounds for the Treatment of Amyotrophic Lateral Sclerosis: An Update. Curr. Neuropharmacol..

[176] Jiang H., Tian X., Guo Y., Duan W., Bu H., Li C. (2011). Activation of Nuclear Factor Erythroid 2-Related Factor 2 Cytoprotective Signaling by Curcumin Protect Primary Spinal Cord Astrocytes against Oxidative Toxicity. Biol. Pharm. Bull..

[177] Wang R., Li Y.-H., Xu Y., Li Y.-B., Wu H.-L., Guo H., Zhang J.-Z., Zhang J.-J., Pan X.-Y., Li X.-J. (2010). Curcumin Produces Neuroprotective Effects via Activating Brain-Derived Neurotrophic Factor/TrkB-Dependent MAPK and PI-3K Cascades in Rodent Cortical Neurons. Prog. Neuropsychopharmacol. Biol. Psychiatry.

[178] Chen H., Li Z., Xu J., Zhang N., Chen J., Wang G., Zhao Y. (2023). Curcumin Induces Ferroptosis in Follicular Thyroid Cancer by Upregulating HO-1 Expression. Oxidative Med. Cell. Longev..

[179] Meng P., Yang R., Jiang F., Guo J., Lu X., Yang T., He Q. (2021). Molecular Mechanism of Astragaloside IV in Improving Endothelial Dysfunction of Cardiovascular Diseases Mediated by Oxidative Stress. Oxidative Med. Cell. Longev..

[180] Huang X.-P., Qiu Y.-Y., Wang B., Ding H., Tang Y.-H., Zeng R., Deng C.-Q. (2014). Effects of Astragaloside IV Combined with the Active Components of Panax Notoginseng on Oxidative Stress Injury and Nuclear Factor-Erythroid 2-Related Factor 2/Heme Oxygenase-1 Signaling Pathway after Cerebral Ischemia-Reperfusion in Mice. Pharmacogn. Mag..

[181] Kontush A., Mann U., Arlt S., Ujeyl A., Lührs C., Müller-Thomsen T., Beisiegel U. (2001). Influence of Vitamin E and C Supplementation on Lipoprotein Oxidation in Patients with Alzheimer’s Disease. Free Radic. Biol. Med..

[182] Mudgal R., Sharma S., Singh S., Ravichandiran V. (2023). The Neuroprotective Effect of Ascorbic Acid against Imidacloprid-Induced Neurotoxicity and the Role of HO-1 in Mice. Front. Neurol..

[183] Zhang N., Zhao W., Hu Z.-J., Ge S.-M., Huo Y., Liu L.-X., Gao B.-L. (2021). Protective Effects and Mechanisms of High-Dose Vitamin C on Sepsis-Associated Cognitive Impairment in Rats. Sci. Rep..

[184] Littlejohns T.J., Henley W.E., Lang I.A., Annweiler C., Beauchet O., Chaves P.H.M., Fried L., Kestenbaum B.R., Kuller L.H., Langa K.M. (2014). Vitamin D and the Risk of Dementia and Alzheimer Disease. Neurology.

[185] Saad El-Din S., Rashed L., Medhat E., Emad Aboulhoda B., Desoky Badawy A., Mohammed ShamsEldeen A., Abdelgwad M. (2020). Active Form of Vitamin D Analogue Mitigates Neurodegenerative Changes in Alzheimer’s Disease in Rats by Targeting Keap1/Nrf2 and MAPK-38p/ERK Signaling Pathways. Steroids.

[186] Liu S., Li G., Tang H., Pan R., Wang H., Jin F., Yan X., Xing Y., Chen G., Fu Y. (2019). Madecassoside Ameliorates Lipopolysaccharide-Induced Neurotoxicity in Rats by Activating the Nrf2-HO-1 Pathway. Neurosci. Lett..

[187] Arab H., Mahjoub S., Hajian-Tilaki K., Moghadasi M. (2016). The Effect of Green Tea Consumption on Oxidative Stress Markers and Cognitive Function in Patients with Alzheimer’s Disease: A Prospective Intervention Study. Casp. J. Intern. Med..

[188] Na H.-K., Kim E.-H., Jung J.-H., Lee H.-H., Hyun J.-W., Surh Y.-J. (2008). (−)-Epigallocatechin Gallate Induces Nrf2-Mediated Antioxidant Enzyme Expression via Activation of PI3K and ERK in Human Mammary Epithelial Cells. Arch. Biochem. Biophys..

[189] Zhou H., Mao Z., Zhang X., Li R., Yin J., Xu Y. (2023). Neuroprotective Effect of Mangiferin against Parkinson’s Disease through G-Protein-Coupled Receptor-Interacting Protein 1 (GIT1)-Mediated Antioxidant Defense. ACS Chem. Neurosci..

[190] Gold-Smith F., Fernandez A., Bishop K. (2016). Mangiferin and Cancer: Mechanisms of Action. Nutrients.

[191] Dillon J.C., Phuc A.P., Dubacq J.P. (1995). Nutritional Value of the Alga Spirulina. World Rev. Nutr. Diet..

[192] Gershwin M.E., Amha B. (2007). Spirulina in Human Nutrition and Health.

[193] Padyana A.K., Bhat V.B., Madyastha K.M., Rajashankar K.R., Ramakumar S. (2001). Crystal Structure of a Light-Harvesting Protein C-Phycocyanin from *Spirulina platensis*. Biochem. Biophys. Res. Commun..

[194] Terry M.J., Maines M.D., Lagarias J.C. (1993). Inactivation of Phytochrome- and Phycobiliprotein-Chromophore Precursors by Rat Liver Biliverdin Reductase. J. Biol. Chem..

[195] McCarty M.F. (2007). Clinical Potential of Spirulina as a Source of Phycocyanobilin. J. Med. Food.

[196] McCarty M.F., Hendler S.S., Rorvik D.M., Inoguchi T. (2010). Compositions for Inhibiting NADPH Oxidase Activity. https://patents.google.com/patent/US20100172971A1/en.

[197] Li Y. (2022). The Bioactivities of Phycocyanobilin from Spirulina. J. Immunol. Res..

[198] Strasky Z., Zemankova L., Nemeckova I., Rathouska J., Wong R.J., Muchova L., Subhanova I., Vanikova J., Vanova K., Vitek L. (2013). *Spirulina platensis* and Phycocyanobilin Activate Atheroprotective Heme Oxygenase-1: A Possible Implication for Atherogenesis. Food Funct..

[199] Zheng J., Inoguchi T., Sasaki S., Maeda Y., McCarty M.F., Fujii M., Ikeda N., Kobayashi K., Sonoda N., Takayanagi R. (2013). Phycocyanin and Phycocyanobilin from *Spirulina platensis* Protect against Diabetic Nephropathy by Inhibiting Oxidative Stress. Am. J. Physiol. -Regul. Integr. Comp. Physiol..

[200] El-Sayed E.-S.M., Hikal M.S., Abo El-Khair B.E., El-Ghobashy R.E., El-Assar A.M. (2018). Hypoglycemic and Hypolipidemic Effects of *Spirulina platensis*, Phycocyanin, Phycocyanopeptide and Phycocyanobilin on Male Diabetic Rats. Arab. Univ. J. Agric. Sci..

[201] Koníčková R., Vaňková K., Vaníková J., Vánová K., Muchová L., Subhanová I., Zadinová M., Zelenka J., Dvořák A., Kolář M. (2014). Anti-Cancer Effects of Blue-Green Alga *Spirulina platensis*, a Natural Source of Bilirubin-like Tetrapyrrolic Compounds. Ann. Hepatol..

[202] Hussein N., Ebied S., Saleh M. (2021). Evaluation of the Anticancer Effect of Violacein, Phycocyanin and Phycocyanobilin on Apoptotic Genes Expression and Glycan Profiles in Breast Cancer Cells. Int. J. Cancer Biomed. Res..

[203] Liu J., Zhang Q.-Y., Yu L.-M., Liu B., Li M.-Y., Zhu R.-Z. (2015). Phycocyanobilin Accelerates Liver Regeneration and Reduces Mortality Rate in Carbon Tetrachloride-Induced Liver Injury Mice. World J. Gastroenterol..

[204] Trotta T., Porro C., Cianciulli A., Panaro M.A. (2022). Beneficial Effects of Spirulina Consumption on Brain Health. Nutrients.

[205] Cervantes-Llanos M., Lagumersindez-Denis N., Marín-Prida J., Pavón-Fuentes N., Falcon-Cama V., Piniella-Matamoros B., Camacho-Rodríguez H., Fernández-Massó J.R., Valenzuela-Silva C., Raíces-Cruz I. (2018). Beneficial Effects of Oral Administration of C-Phycocyanin and Phycocyanobilin in Rodent Models of Experimental Autoimmune Encephalomyelitis. Life Sci..

[206] Pavón-Fuentes N., Marín-Prida J., Llópiz-Arzuaga A., Falcón-Cama V., Campos-Mojena R., Cervantes-Llanos M., Piniella-Matamoros B., Pentón-Arias E., Pentón-Rol G. (2020). Phycocyanobilin Reduces Brain Injury after Endothelin-1-Induced Focal Cerebral Ischaemia. Clin. Exp. Pharmacol. Physiol..

[207] Pentón-Rol G., Marín-Prida J., Falcón-Cama V. (2018). C-Phycocyanin and Phycocyanobilin as Remyelination Therapies for Enhancing Recovery in Multiple Sclerosis and Ischemic Stroke: A Preclinical Perspective. Behav. Sci..

[208] McCarty M.F., Barroso-Aranda J., Contreras F. (2010). Oral Phycocyanobilin May Diminish the Pathogenicity of Activated Brain Microglia in Neurodegenerative Disorders. Med. Hypotheses.

[209] Chamorro G., Pérez-Albiter M., Serrano-García N., Mares-Sámano J.J., Rojas P. (2006). Spirulina Maxima Pretreatment Partially Protects against 1-Methyl-4-Phenyl-1,2,3,6-Tetrahydropyridine Neurotoxicity. Nutr. Neurosci..

[210] Marín-Prida J., Pavón-Fuentes N., Lagumersindez-Denis N., Camacho-Rodríguez H., García-Soca A.M., Sarduy-Chávez R.d.l.C., Vieira L.M., Carvalho-Tavares J., Falcón-Cama V., Fernández-Massó J.R. (2022). Anti-Inflammatory Mechanisms and Pharmacological Actions of Phycocyanobilin in a Mouse Model of Experimental Autoimmune Encephalomyelitis: A Therapeutic Promise for Multiple Sclerosis. Front. Immunol..

[211] Gardón D.P., Cervantes-Llanos M., Matamoros B.P., Rodríguez H.C., Tan C.-Y., Marín-Prida J., Falcón-Cama V., Pavón-Fuentes N., Lemus J.G., Ruiz L.d.l.C.B. (2022). Positive Effects of Phycocyanobilin on Gene Expression in Glutamate-Induced Excitotoxicity in SH-SY5Y Cells and Animal Models of Multiple Sclerosis and Cerebral Ischemia. Heliyon.

[212] Pendyala B., Patras A., Dash C. (2021). Phycobilins as Potent Food Bioactive Broad-Spectrum Inhibitors Against Proteases of SARS-CoV-2 and Other Coronaviruses: A Preliminary Study. Front. Microbiol..

[213] Chen Y.-H., Chang G.-K., Kuo S.-M., Huang S.-Y., Hu I.-C., Lo Y.-L., Shih S.-R. (2016). Well-Tolerated Spirulina Extract Inhibits Influenza Virus Replication and Reduces Virus-Induced Mortality. Sci. Rep..

[214] Teas J., Hebert J.R., Fitton J.H., Zimba P.V. (2004). Algae—A Poor Man’s HAART?. Med. Hypotheses.

[215] Ngo-Matip M.-E., Pieme C.A., Azabji-Kenfack M., Moukette B.M., Korosky E., Stefanini P., Ngogang J.Y., Mbofung C.M. (2015). Impact of Daily Supplementation of *Spirulina platensis* on the Immune System of Naïve HIV-1 Patients in Cameroon: A 12-Months Single Blind, Randomized, Multicenter Trial. Nutr. J..

[216] Teas J., Irhimeh M.R. (2012). Dietary Algae and HIV/AIDS: Proof of Concept Clinical Data. J. Appl. Phycol..

[217] Nihal B., Gupta N.V., Gowda D.V., Manohar M. (2018). Formulation and Development of Topical Anti Acne Formulation of Spirulina Extract. Int. J. Appl. Pharm..

[218] Mapoung S., Arjsri P., Thippraphan P., Semmarath W., Yodkeeree S., Chiewchanvit S., Piyamongkol W., Limtrakul P. (2020). Photochemoprotective Effects of *Spirulina platensis* Extract against UVB Irradiated Human Skin Fibroblasts. South. Afr. J. Bot..

[219] Ragusa I., Nardone G.N., Zanatta S., Bertin W., Amadio E. (2021). Spirulina for Skin Care: A Bright Blue Future. Cosmetics.

[220] Reportlinker Global Market Study on Spirulina: Powder Product form Segment Anticipated to Dominate the Global Market in Terms of both Value and Volume during 2016–2026. https://www.prnewswire.com/news-releases/global-market-study-on-spirulina-powder-product-form-segment-anticipated-to-dominate-the-global-market-in-terms-of-both-value-and-volume-during-2016---2026-300443004.html.

[221] Qin X. (2008). Bilirubin Would Be the Indispensable Component for Some of the Most Important Therapeutic Effects of Calculus Bovis (Niuhuang). Chin. Med. J..

[222] Yu Z.-J., Xu Y., Peng W., Liu Y.-J., Zhang J.-M., Li J.-S., Sun T., Wang P. (2020). Calculus Bovis: A Review of the Traditional Usages, Origin, Chemistry, Pharmacological Activities and Toxicology. J. Ethnopharmacol..

[223] Zhang S., Jiang X., Wang Y., Lin K., Zhang Z., Zhang Z., Zhu P., Ng M.L., Qu S., Sze S.C.W. (2021). Protective Effect of An-Gong-Niu-Huang Wan Pre-Treatment Against Experimental Cerebral Ischemia Injury via Regulating GSK-3β/HO-1 Pathway. Front. Pharmacol..

[224] Banjerdpongchai R., Wudtiwai B., Khawon P. (2016). Induction of Human Hepatocellular Carcinoma HepG2 Cell Apoptosis by Naringin. Asian Pac. J. Cancer Prev..

[225] Martins T., Barros A.N., Rosa E., Antunes L. (2023). Enhancing Health Benefits through Chlorophylls and Chlorophyll-Rich Agro-Food: A Comprehensive Review. Molecules.

[226] Mishra V.K., Bachheti R., Husen A. (2011). Medicinal Uses of Chlorophyll: A Critical Overview. Chlorophyll: Structure, Production and Medicinal Uses.

[227] Ferruzzi M.G., Blakeslee J. (2007). Digestion, Absorption, and Cancer Preventative Activity of Dietary Chlorophyll Derivatives. Nutr. Res..

[228] Vaňková K., Marková I., Jašprová J., Dvořák A., Subhanová I., Zelenka J., Novosádová I., Rasl J., Vomastek T., Sobotka R. (2018). Chlorophyll-Mediated Changes in the Redox Status of Pancreatic Cancer Cells Are Associated with Its Anticancer Effects. Oxid. Med. Cell Longev..

[229] Pittala V., Vanella L., Salerno L., Di Giacomo C., Acquaviva R., Raffaele M., Romeo G., Modica M.N., Prezzavento O., Sorrenti V. (2017). Novel Caffeic Acid Phenethyl Ester (Cape) Analogues as Inducers of Heme Oxygenase-1. Curr. Pharm. Des..

[230] Šmíd V., Šuk J., Kachamakova-Trojanowska N., Jašprová J., Valášková P., Józkowicz A., Dulak J., Šmíd F., Vítek L., Muchová L. (2018). Heme Oxygenase-1 May Affect Cell Signalling via Modulation of Ganglioside Composition. Oxid. Med. Cell Longev..

[231] Moon S., Kim C.-H., Park J., Kim M., Jeon H.S., Kim Y.-M., Choi Y.K. (2022). Induction of BVR-A Expression by Korean Red Ginseng in Murine Hippocampal Astrocytes: Role of Bilirubin in Mitochondrial Function via the LKB1–SIRT1–ERRα Axis. Antioxidants.

[232] Wu L.-X., Guo C.-X., Qu Q., Yu J., Chen W.-Q., Wang G., Fan L., Li Q., Zhang W., Zhou H.-H. (2012). Effects of Natural Products on the Function of Human Organic Anion Transporting Polypeptide 1B1. Xenobiotica.

[233] Vítek L., Carey M.C. (2003). Enterohepatic Cycling of Bilirubin as a Cause of “black” Pigment Gallstones in Adult Life. Eur. J. Clin. Investig..

[234] Vítek L., Zelenka J., Zadinová M., Malina J. (2005). The Impact of Intestinal Microflora on Serum Bilirubin Levels. J. Hepatol..

[235] Hall B., Levy S., Dufault-Thompson K., Ndjite G.M., Weiss A., Braccia D., Jenkins C., Yang Y., Arp G., Abeysinghe S. (2023). Discovery of the Gut Microbial Enzyme Responsible for Bilirubin Reduction to Urobilinogen. bioRxiv.

